# Human giant congenital melanocytic nevus exhibits potential proteomic alterations leading to melanotumorigenesis

**DOI:** 10.1186/1477-5956-10-50

**Published:** 2012-08-20

**Authors:** Hyoung Kyu Kim, Yong Kyu Kim, In-Sung Song, Sung-Ryul Lee, Seung Hun Jeong, Min Hee Kim, Dae Yun Seo, Nari Kim, Byoung Doo Rhee, Kyoung Soo Ko, Kwan Chul Tark, Chul Gyoo Park, Je-Yoel Cho, Jin Han

**Affiliations:** 1 National Research Laboratory for Mitochondrial Signaling, Department of Physiology, College of Medicine, Cardiovascular and Metabolic Disease Center, Inje University, Busan, Korea; 2 Apgujung YK Plastic Surgery Center, Seoul, Korea; 3 Department of Plastic and Reconstructive Surgery, College of Medicine, Yonsei University, Seoul, Korea; 4 Department of Plastic and Reconstructive Surgery, National Medical Center, Seoul, Korea; 5 Department of Veterinary Biochemistry, College of Veterinary Medicine, Seoul National University, Seoul, 151-742, Korea

**Keywords:** Giant congenital melanocytic nevi, Melanotumorigenesis, Proteomics, 14-3-3 epsilon, 14-3-3 tau, Systemic analysis

## Abstract

**Background:**

A giant congenital melanocytic nevus (GCMN) is a malformation of the pigment cells. It is a distress to the patients for two reasons: one is disfigurement, and the other is the possibility of malignant changes. However, the underlying mechanisms of the development of GCMN and melanotumorigenesis in GCMN are unknown. Hence, the aim of this study was to identify the proteomic alterations and associated functional pathways in GCMN.

**Results:**

Proteomic differences between GCMN (n = 3) and normal skin samples (n = 3) were analyzed by one-dimensional-liquid chromatography-tandem mass spectrometry Relative levels of the selected proteins were validated using western blot analysis. The biological processes associated with the abundance modified proteins were analyzed using bioinformatic tools. Among the 46 abundance modified proteins, expression of 4 proteins was significantly downregulated and expression of 42 proteins was significantly upregulated in GCMN compared to normal skin samples (p < 0.05). More importantly, 31% of the upregulated proteins were implicated in various cancers, with five proteins being specifically related with melanoma. The abundance modified proteins in GCMN were involved in the biological processes of neurotrophin signaling, melanosome, and downregulated of MTA-3 in ER-negative breast tumors. In particular, an increase in the expression of the 14-3-3 protein family members appeared to be associated with key cellular biological functions in GCMN. Western blot analysis confirmed the upregulation of 14-3-3epsilon, 14-3-3 tau, and prohibitin in GCMN.

**Conclusion:**

These findings suggest that GCMN exhibits potential proteomic alterations, which may play a role in melanotumorigenesis, and the significant alteration of 14-3-3 family proteins could be a key regulator of the biological pathway remodeling in GCMN.

## Background

Congenital melanocytic nevi (CMN) are pigment cell malformations that are visible at birth, or are nevi showing congenital features that become clinically obvious shortly after birth [1]. CMN is caused by abnormal melanocyte differentiation, migration, and deposition in the dermis during the early stages of embryogenesis [2,3]. It is a distress to patients for two reasons: one is disfigurement, and the other is the increased risk of developing malignant melanoma [3,4], especially in individuals with giant congenital melanocytic nevi (GCMN; over 20 cm in diameter) [5]. Several genomic and proteomic studies have been performed to elucidate the mechanism of melanotumorigenesis arising from CMN. Gene-based analyses have revealed that the oncogenic *BRAF*[6] and *NRAS*[7] mutations are frequently seen in CMN. Additionally, increased Bcl-2 expression in CMN has been suggested to suppress apoptosis, which otherwise plays an important role in the maintenance of nevocytes [8]. In spite of these findings, the major biological processes and pathways of melanotumorigenesis remain unclear. Therefore, the complete proteomic characterization of GCMN and related biological pathways through comparative protein profiling is essential to understand the underlying mechanism of the origin of GCMN and the subsequent process of melanotumorigenesis.

In this study, the proteomic alteration and systemic properties of GCMN were assessed, with the aim of gaining an insight into the functional association between GCMN and melanotumorigenesis. We used label-free liquid chromatography-mass spectrometry (LC-MS) and established bioinformatic tools to identify the proteins that may play a key role in the malignant transformation of GCMN. We found that the 46 proteins were changed in protein abundance levels between normal skin and GCMN samples, and these proteins belonged to tightly organized functional clusters. Moreover, we found that five of the identified proteins were implicated in melanoma. The results of this study will improve our understanding in the biological identification of GCMN and the possible mechanisms that give rise to GCMN-associated melanotumorigenesis.

## Results

### Characteristics of the study population

**Figure 1:**
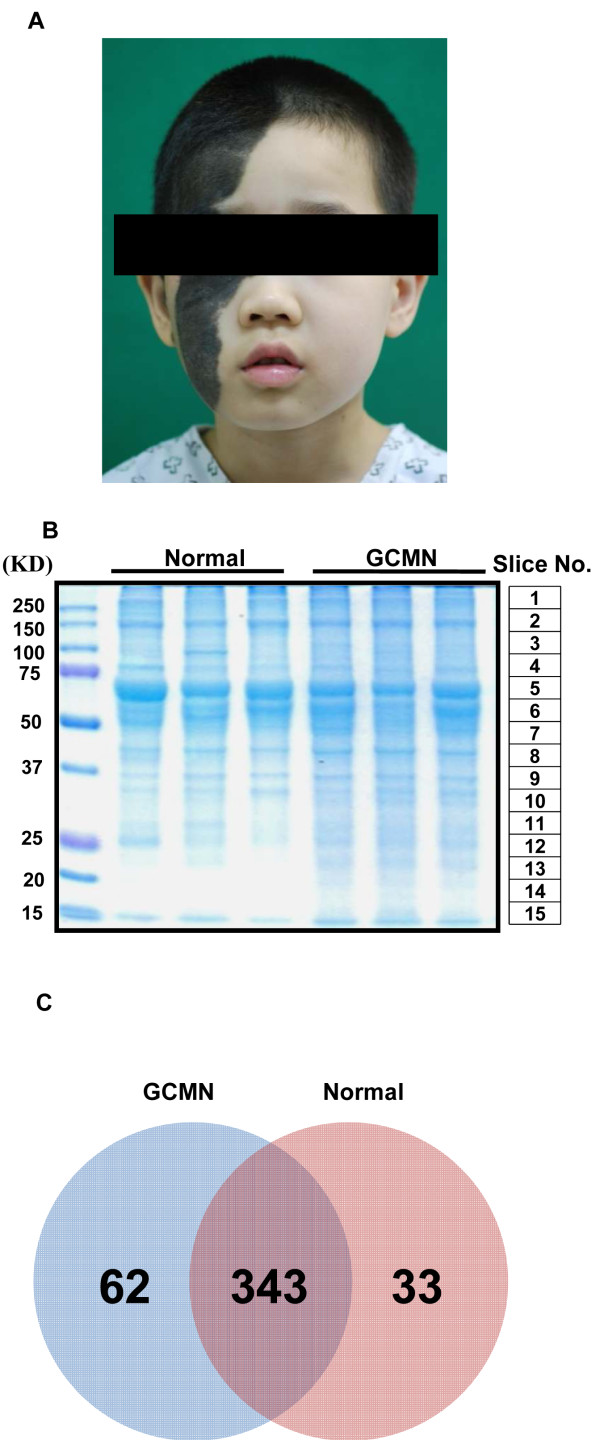
**Comparative proteomic analysis of giant congenital melanocytic nevi (GCMN) and normal skin samples using one-dimensional-liquid chromatography-electrospray ionization-tandem mass spectrometry.** (**A**) A representative GCMN lesion of a donor patient (**B**) Coomassie-stained gel of normal skin and GCMN proteins (n = 3 each). Slice number indicates the matching mass peak result in Additional file 1: Figure S1. (**C**) Venn diagram of the identified proteins in normal and GCMN skin samples.

The clinical characteristics of patients who provided GCMN and normal skin samples are listed in Table 1. All GCMN were over 20 cm in diameter. A representative GCMN lesion in a patient is shown in Figure 1A. To minimize environmental bias between individuals, we collected three paired normal and GCMN skin samples from three GCMN donors for one-dimensional-liquid chromatography-tandem mass spectrometry (1D-LC-MS/MS) analysis (Table 1). Seven unpaired samples from normal skin and GCMN were used for western blot analysis to validate the proteomic results. The average age of GCMN patients who donated samples for western blot analysis was lower (5.57 ± 1.90) than that of the normal skin donors (22.71 ± 11.01, p = 0.0058, n = 7).

**Table 1:** Clinical characteristics of GCMN and normal skin samples

**Sample ID**	**Age, yr**	**Gender**	**Location**	**Experiment**
***Normal***				
Normal 1*	8	Male	Face	LC-MS
Normal 2*	5	Female	Face	LC-MS
Normal 3*	6	Female	Face	LC-MS
Normal 4	14	Female	Forehead	Western blot
Normal 5	11	Female	Face	Western blot
Normal 6	19	Female	Forehead	Western blot
Normal 7	36	Female	Face	Western blot
Normal 8	18	Female	Face	Western blot
Normal 9	21	Female	Face	Western blot
Normal 10	40	Female	Face	Western blot
***GCMN***				
GCMN 1	8	Male	Face	LC-MS
GCMN 2	5	Female	Face	LC-MS
GCMN 3	6	Female	Face	LC-MS
GCMN 4	8	Male	Forehead	Western blot
GCMN 5	6	Female	Abdomen	Western blot
GCMN 6	3	Female	Left leg	Western blot
GCMN 7	3	Male	Face	Western blot
GCMN 8	6	Female	Forehead	Western blot
GCMN 9	7	Male	Abdomen	Western blot
GCMN 10	6	Female	Head	Western blot

* Normal skin samples excised from GCMN patient ID 1, 2, 3.

### Proteomic alterations in GCMN

**Figure 2:**
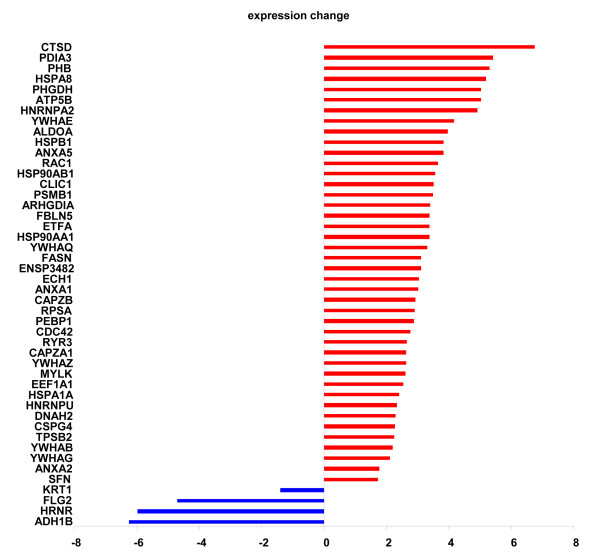
**Fold-changes in abundance modified proteins in GCMN compared to normal skin.** The histogram displays the protein symbol and the averaged fold-change of each differentially expressed protein in GCMN. A total of 46 proteins were differentially expressed, with 4 proteins downregulated and 42 upregulated in GCMN compared to normal skin.

The same amount (20 μg) of soluble proteins obtained from GCMN and normal skin samples were analyzed on sodium dodecyl sulfate polyacrylamide gel electrophoresis (SDS-PAGE; Figure 1B). The Coomassie blue-stained gels were sliced into 15 uniform slices. The total ion current chromatogram of each slice was acquired (Additional file 1: Figure S1), which led to the identification of 438 non-redundant proteins (Additional file 2: Table S2). Among these, 343 proteins were commonly identified in both the groups, 62 proteins were detected only in GCMN samples, and 33 proteins were detected only in normal skin samples (Figure 1C). Forty-six abundance modified proteins were identified whose protein abundance level was significantly (Student’s t-test with Bonferroni correction, p < 0.05) different between GCMN and normal skin (Figure 1D). The large majority of these abundance modified proteins were upregulated (42 of 46, 91.3%), and very few (4 proteins, 8.7%) were downregulated in GCMN (Figure 2, Table 2). The most upregulated protein in GCMN was cathepsin D (CTSD) [GenBank Gene ID: 1509], which was expressed at a 6.8-fold higher level than in normal skin. In addition, protein disulfide-isomerase A3 precursor (PDIA3, 5.4-fold) [GenBank Gene ID: 2923], prohibitin (PHB, 5.3-fold) [GenBank Gene ID: 5245], heat-shock protein 70 (5.2-fold) [GenBank Gene ID: 3312], D-3-phosphoglycerate dehydrogenase (5-fold) [GenBank Gene ID: 26227], and adenosine-5'-triphosphate synthase subunit beta (5-fold) [GenBank Gene ID: 506] were highly (>4-fold) upregulated in GCMN. The 4 proteins downregulated in GCMN were cytokeratin-1 (−1.4-fold) [GenBank Gene ID: 3848], filaggrin 2 (−4.7-fold) [GenBank Gene ID: 388698], hornerin (−6.0-fold) [GenBank Gene ID: 388697], and alcohol dehydrogenase 1B (−6.2-fold) [GenBank Gene ID: 125]. Interestingly, we found that 33% (14 of total 42) of the significantly upregulated proteins in GCMN samples were implicated in various kinds of cancers (Table 2). Five of the 14 proteins, namely CTSD [GenBank Gene ID: 1509][9,10], PHB [GenBank Gene ID: 5245][11,12], chondroitin sulfate proteoglycan 4 [GenBank Gene ID: 1464] [13], phosphatidylethanolamine-binding protein 1 [GenBank Gene ID: 5037] [14], and ribosomal protein SA [GenBank Gene ID: 3921] [15] were related with melanoma. 

**Table 2:** Abundance modified proteins in GCMN

**Identified proteins**	**Cancer implication**	**Accession number**^1^	**p-Value**	**Fold-change**	GeneID^2^
***Post-translational modification, protein turnover, chaperones (15)***					
CTSD cathepsin D -	breast [52] ovarian [53] melanoma [14,15]	IPI00011229	0.024	6.8	1509
HSP90AA1 heat shock protein 90-kDa alpha (cytosolic), class A member 1 isoform 1		IPI00382470	0.02	3.4	3320
HSP90AB1 heat shock protein HSP 90-beta		IPI00414676	0.013	3.6	3326
HSPA1A;HSPA1B heat shock 70-kDa protein 1		IPI00304925	0.031	2.4	3303
HSPA8 Isoform 1 of heat shock cognate 71-kDa protein		IPI00003865	0.0016	5.2	3312
HSPB1 heat shock protein beta-1		IPI00025512	0.0027	3.8	3315
PDIA3 protein disulfide-isomerase A3	ovarian [54]	IPI00025252	0.012	5.4	2923
PHB prohibitin	gastric carcinoma [28], thyroid cancer [29], hepatocellular carcinoma [30], melanoma [16,17]	IPI00017334	0.025	5.3	5245
PSMB1 proteasome subunit beta type-1		IPI00025019	0.045	3.5	5689
SFN Isoform 1 of 14-3-3 protein sigma		IPI00013890	0.027	1.7	2810
YWHAB long isoform of 14-3-3beta/alpha		IPI00216318	0.0012	2.2	7529
YWHAE 14-3-3epsilon		IPI00000816	0.0051	4.2	7531
YWHAG 14-3-3gamma		IPI00220642	0.024	2.1	7532
YWHAQ 14-3-3theta		IPI00018146	0.00088	3.3	10971
YWHAZ 14-3-3zeta/delta		IPI00021263	0.0021	2.6	7534
***Cytoskeleton (4)***					
CAPZA1 F-actin-capping protein subunit alpha-1		IPI00005969	0.0044	2.6	829
CAPZB cDNA, FLJ93598, highly similar to *Homo sapiens* capping protein (actin filament) muscle Z-line, beta		IPI00641107	0.017	2.9	832
Isoform 2 of dynein heavy chain 2, axonemal		IPI00651691	0.004	2.3	146754
Isoform 2 of myosin light chain kinase, smooth muscle		IPI00221255	0.0018	2.6	4638
***General function prediction only (5)***					
CDC42 Isoform 2 of cell-division control protein 42 homolog	immune escape of cancer [55]	IPI00016786	0.017	2.8	998
CSPG4 chondroitin sulfate proteoglycan 4	melanoma, human carcinoma, sarcoma [18]	IPI00019157	0.026	2.3	1464
HNRNPA2B1 isoform B1 of heterogeneous nuclear ribonucleoproteins A2/B1	glioblastoma[56] lung cancer [57]	IPI00396378	0.015	4.9	3181
PEBP1 phosphatidylethanolamine-binding protein 1	prostate [58], breast [59], gastrointestinal stromal [60], melanoma [19], ovarian [61]	IPI00219446	0.026	2.9	5037
RAC1 isoform A of Ras-related C3 botulinum toxin substrate 1	skin tumor [62]	IPI00010271	0.0092	3.7	5879
***Carbohydrate transport and metabolism (3)***					
ALDOA fructose-bisphosphate aldolase A		IPI00465439	0.034	4	226
Uncharacterized protein ENSP00000348237		IPI00453476	0.0012	3.1	
FLG2 filaggrin-2		IPI00397801	0.000021	0.2	388698
***Energy production and conversion (2)***					
ATP5B ATP synthase subunit beta, mitochondrial		IPI00303476	0.032	5	506
ETFA electron transfer flavoprotein subunit alpha, mitochondrial		IPI00010810	0.0028	3.4	2108
***Signal transduction mechanisms (3)***					
ARHGDIA rho GDP-dissociation inhibitor 1	mesothelioma [63]	IPI00003815	0.047	3.4	396
FBLN5 fibulin-5	breast cancer [64]	IPI00294615	0.0028	3.4	10516
RYR3 uncharacterized protein RYR3		IPI00217185	0.013	2.7	6263
***Intracellular trafficking, secretion, and vesicular transport (3)***					
ANXA1 annexin A1	breast cancer [65]	IPI00218918	0.03	3	301
ANXA2 annexin A2 isoform 1	breast cancer [66]	IPI00418169	0.0059	1.8	302
ANXA5 annexin A5	colorectal cancer [67]	IPI00329801	0.025	3.8	308
***Amino acid transport and metabolism (2)***					
PHGDH D-3-phosphoglycerate dehydrogenase	breast cancer [68]	IPI00011200	0.031	5	26227
TPSB2 TPSB2		IPI00419942	0.043	2.2	64499
***Lipid transport and metabolism (2)***					
ECH1 delta(3,5)-delta(2,4)-dienoyl-CoA isomerase, mitochondrial		IPI00011416	0.00034	3.1	1891
FASN fatty acid synthase	breast cancer, endometrial cancer [69] prostate cancer [70]	IPI00026781	0.023	3.1	2194
***Translation, ribosomal structure, and biogenesis (2)***					
EEF1A1 elongation factor 1-alpha 1		IPI00396485	0.012	2.5	1915
RPSA ribosomal protein SA, 33-kDa protein	melanoma [20]	IPI00413108	0.024	2.9	3921
***RNA processing and modification (1)***					
HNRNPU short isoform of heterogeneous nuclear ribonucleoprotein U		IPI00479217	0.0037	2.3	3192
***Cell cycle control, cell division, chromosome partitioning (1)***					
KRT1 keratin, type II cytoskeletal 1		IPI00220327	0.016	0.7	3848
***Inorganic ion transport and metabolism (1)***					
CLIC1 chloride intracellular channel protein 1		IPI00010896	0.015	3.5	1192
***Secondary metabolites biosynthesis, transport, and catabolism (1)***					
ADH1B alcohol dehydrogenase 1B		IPI00473031	0.025	0.2	125
***Function unknown (1)***					
HRNR hornerin		IPI00398625	0.035	0.2	388697

Accession Number^1^: International Protein Index (IPI) accession number, GeneID^2^: NCBI GeneID.

### Systemic properties of the altered GCMN proteome

**Figure 3:**
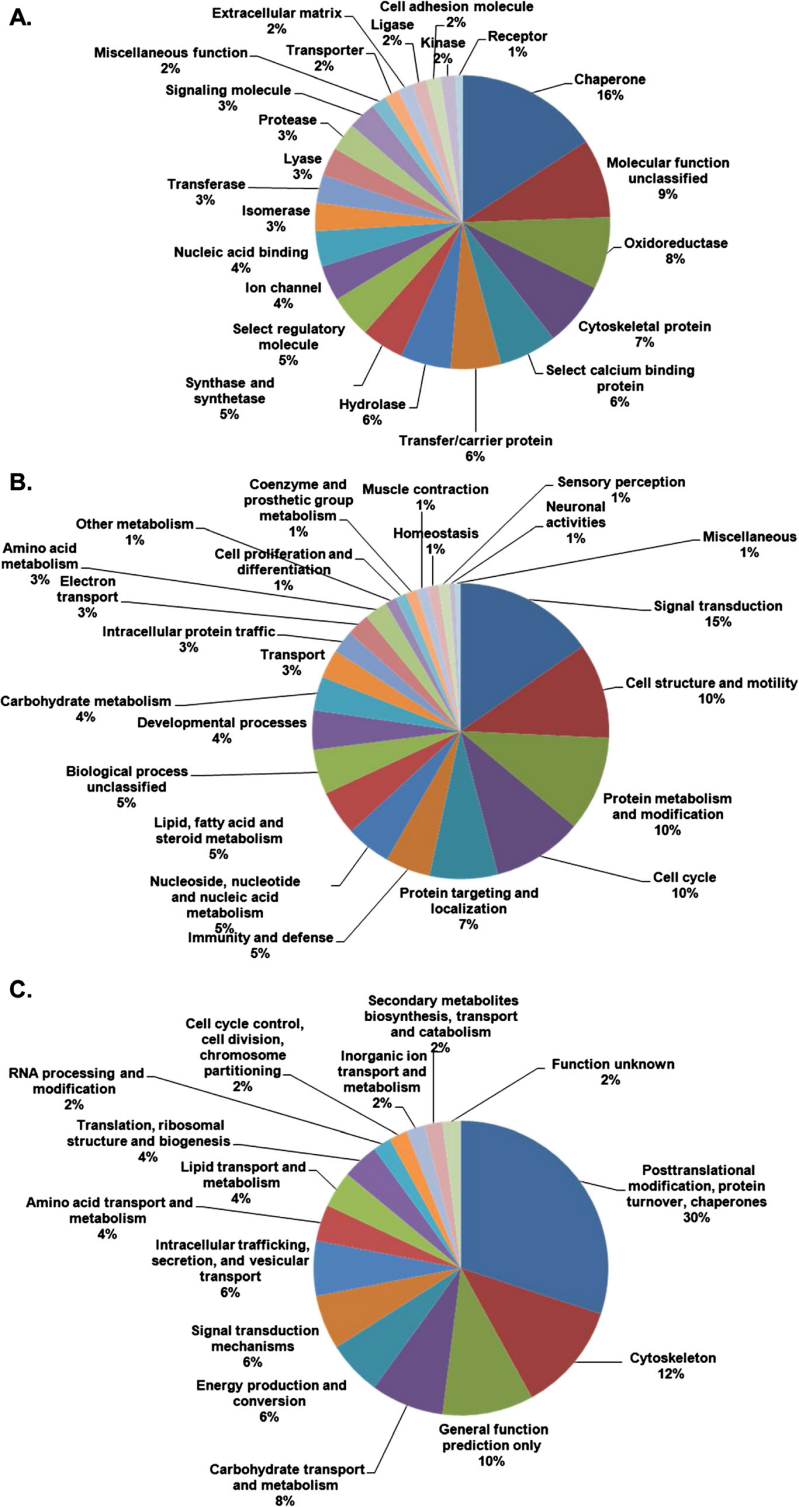
**Functional categories and their corresponding percentages in the GCMN proteome.** Molecular function (**A**), biological process (**B**), and clusters of orthologous groups (COG) (**C**) were analyzed using the online bioinformatic tools PANTHER (http://www.pantherdb.org/) and COGnitor provided by the NCBI database (http://www.ncbi.nlm.nih.gov/COG/old/xognitor.html).

Proteomic profiling of certain diseases has been increasingly used to acquire data on a large number of proteins to identify potential biomarkers and to gain insight into the underlying mechanisms of a variety of diseases. However, the massive amount of generated information often only increases the complexity of these analyses. To address this issue, we developed a systemic approach to analyze the proteomic data generated in the present study. To gain an insight into the systemic properties of the GCMN proteome, the 46 abundance modified proteins were categorized based on molecular function, biological process, and Clusters of Orthologous Groups of proteins (COG) analysis. The proteins were classified into the following classes according to their molecular functions: chaperones (16% of total), oxidoreductases (8%), and cytoskeletal elements (7%) (Figure 3A). Proteins associated with the biological processes of signal transduction (15%), cell structure and motility (10%), protein metabolism and modification (10%), and cell cycle (10%) were markedly altered in their expression. The COG analysis showed that the altered proteins in GCMN samples were involved in posttranslational modification, turnover, chaperones (30%), and the cytoskeleton (12%; Figure 3C), which was consistent with the molecular function and biological process classification.

Further network analysis was carried out using the software tool, ClueGO, to identify enriched functional groups in the GCMN samples and obtain detailed information on each one. Gene Ontology (GO)_biological process, GO_cellular component, GO_molecular function, KEGG pathway, and Reactome_biocarta were selected as ontology sources. The specific functional groups identified as significantly enriched in GCMN were melanosome (GO_cellular component), neurotrophin signaling pathway (KEGG pathway), downregulated of MTA-3 in ER-negative breast tumors (Biocarta), cell cycle (KEGG pathway), phospholipase inhibitor activity (GO_molecular function), and glycolysis/gluconeogenesis (KEGG pathway; Table 3, Additional file 3: Figure S2).

**Table 3:** Enriched functional group in GCMN

**Functional group**	**Group Genes**	**GOID**	**% Genes**^**1**^	**p-Value**^**2**^	**p-Value/B**^**3**^
Cell cycle	ANXA1|SFN|YWHAB|YWHAE|YWHAG|YWHAQ|YWHAZ	*KEGG:04110*	5.0	9.00E-07	4.77E-05
Phospholipase inhibitor activity	ANXA1|ANXA2|ANXA5|ARHGDIA|ATP5B|KRT1	*GO:0004859*	27.3	3.17E-06	1.68E-04
Melanosome	ANXA1|ANXA2|ANXA5|ATP5B|CLIC1|CTSD|FASN|HRNR|HSP90AA1|HSP90AB1|HSPA1B|HSPA8|HSPB1|KRT1|PDIA3|RAC1|SFN|YWHAB|YWHAE|YWHAG|YWHAZ	*GO:004247*	12.8	4.09E-16	2.17E-14
Neurotrophin signaling pathway	ACTB|ARHGDIA|CAPZA1|CAPZB|CDC42|HSPB1|MYLK|RAC1|SFN|YWHAB|YWHAE|YWHAG|YWHAQ|YWHAZ	*KEGG:04722*	6.1	2.36E-09	1.25E-07
Downregulated of MTA-3 in ER-negative breast tumors	ADH1B|ALDOA|CTSD|ECH1|HSPB1|PHGDH|RYR3	*BioCarta:204*	28.6	4.94E-08	2.62E-06
Glycolysis/Gluconeogenesis	ADH1B|ALDOA|ECH1|FASN	*KEGG:00010*	4.7	7.23E-04	0.038

^1^% of associated genes identified in our results/total known genes involved in the function or pathway, ^2^Term p-Value, ^3^Term p-Value with Bonferroni correction.

**Figure 4:**
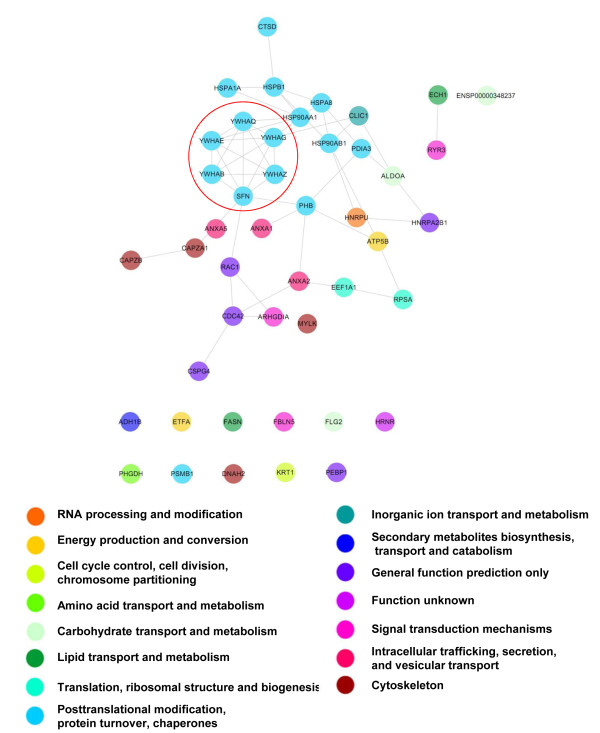
**The proteome network of GCMN.** Interactions between the proteins altered in GCMN were analyzed by String 8.0 and visualized with COG functional clustering. The proteins 14-3-3 sigma (or SFN, 1.7-fold), 14-3-3 beta/alpha (YWHAB, 2.2-fold), 14-3-3 epsilon (YWHAE, 4.2-fold), 14-3-3 gamma (YWHAG, 2.1-fold), 14-3-3 theta (YWHAQ, 3.3-fold), and 14-3-3 zeta/delta (YWHAZ, 2.6-fold) were tightly linked with each other and formed specific functional clusters (red circles).

Because proteins rarely act alone, but rather interact as a group, thus comprising a functional cluster, a protein-protein interacting network was constructed using STRING 8.0. This network consisted of 45 of the 46 abundance modified proteins, with 52 interactions between them. In the GCMN network, the upregulated proteins were tightly linked with each other, forming a large cluster. The biological meaning of the cluster was determined by overlaying the COG of each protein on the network, which revealed shared COGs between close neighboring proteins (Figure 4). Moreover, the GCMN network contained a highly specific protein cluster, identified as the 14-3-3 protein family, whose members were expressed at significantly higher levels than in normal skin as follows: 14-3-3 sigma (or SFN, 1.7-fold) [GenBank Gene ID: 2810], 14-3-3 protein beta/alpha (YWHAB, 2.2-fold) [GenBank Gene ID: 7529], 14-3-3 protein epsilon (YWHAE, 4.2-fold) [GenBank Gene ID: 7531], 14-3-3 protein gamma (YWHAG, 2.1-fold) [GenBank Gene ID: 7532], 14-3-3 protein theta (YWHAQ, 3.3-fold) [GenBank Gene ID: 10971], and 14-3-3 protein zeta/delta (YWHAZ, 2.6-fold) [GenBank Gene ID: 7534].

### Comparison of systemic properties between GCMN and metastatic melanoma

**Figure 5:**
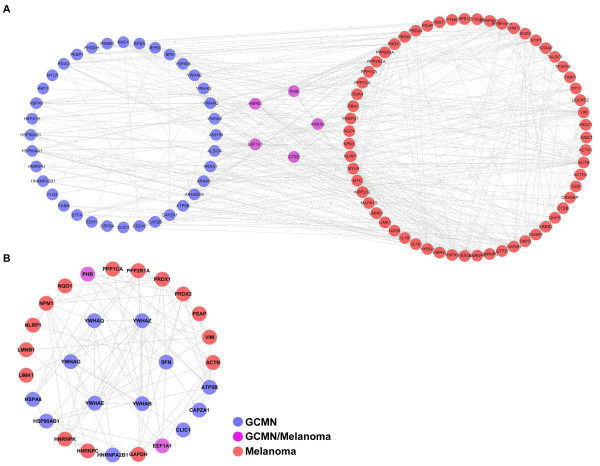
**Protein-protein interaction map of GCMN and melanoma. A**. GCMN and melanoma show five common upregulated proteins, namely PHB, HSPB1, ANXA2, CTSD, and EEF1A1. There are 217 cross-interactions between the GCMN and melanoma proteome. **B**. A total of 23 proteins (6 proteins from the GCNM proteome and 17 proteins from the melanoma proteome) interact with the differentially expressed 14-3-3 protein family in GCMN. Two interacting proteins, PHB and EEF1A1, were co-detected in both groups.

To investigate the inter-relationship between the proteomic characteristics of GCMN and melanoma, we compared our GCMN proteome network with a recently reported proteome of metastatic melanoma cell line that contained 110 non-redundant proteins [16]. We selected 63 of the total melanoma proteins that had been included in the two high-score biological networks and analyzed protein-protein interactions between the GCMN and melanoma proteomes (Figure 5A). Five proteins including PHB, CTSD, elongation factor 1-alpha 1 (EEF1A1), Annexin A2 (ANXA2), and heat-shock protein beta-1 (HSPB1) were similarly upregulated in both proteome sets. Subsequently, we found that 217 interactions existed between the GCMN and melanoma proteomes. To estimate the biological importance of the 14-3-3 family proteins in the process of melanotumorigenesis, we next analyzed the protein-protein interactions of 14-3-3 family proteins with the GCMN and melanoma proteomes. We identified a total of 23 proteins, out of which 6 proteins in the GCNM and 17 proteins in the melanoma proteome possibly interacted with the 14-3-3 family proteins (Figure 5B). Two proteins interacting with 14-3-3, PHB and EEF1A1, were co-detected in both GCMN and melanoma proteomes. Additionally, the average interaction number of the 14-3-3 family proteins (7.66) was 2-fold higher than the average interaction number of other proteins (3.80). These results suggest that there are similar proteomic alterations in both GCMN and metastatic melanoma and the 14-3-3 family proteins may play an important role in melanotumorigenesis and tumor progression. 

### Validation of the abundance modified proteins

**Figure 6:**
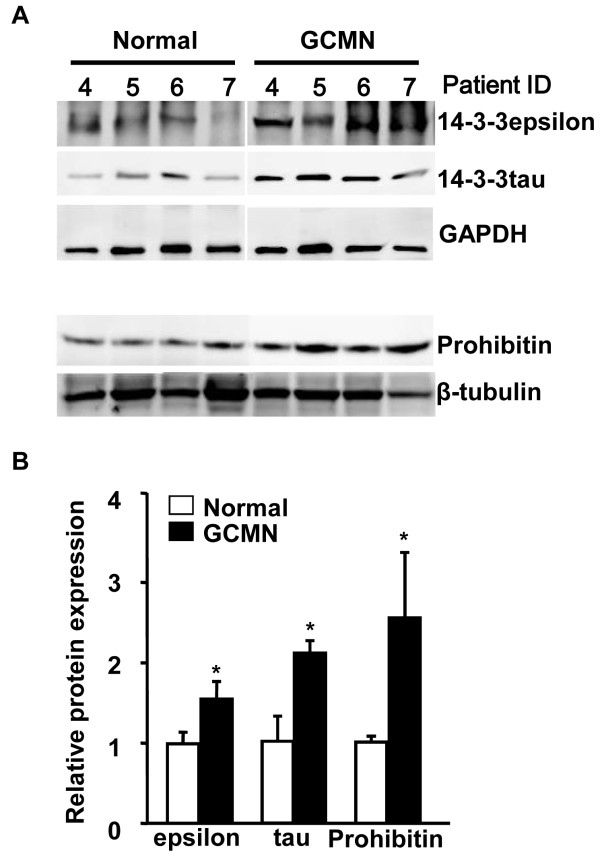
**Validation of protein expression of the 14-3-3 protein family and PHB. A**. Representative western blot images of 14-3-3 epsilon, 14-3-3 tau, and prohibitin proteins in normal and GCMN skin samples. **B**. The relative protein expression of 14-3-3 epsilon, 14-3-3 tau, and prohibitin was significantly increased in GCMN samples compared to normal skin samples (n = 7). *p < 0.05, two-tailed unpaired Student’s t-test *vs.* normal skin sample.

To further validate the proteomic results, we evaluated the altered expression of 14-3-3 alpha/beta, sigma, epsilon, zeta, tau, and of prohibitin in GCMN and unpaired normal skin samples (n = 7) using semi-quantitative western blots. The integrated values of each protein band are reported in supplementary Additional files 4: Table S2 and Additional files 5.: Table S3 Because it was difficult to obtain skin specimens from normal children younger than 10 years of age, we could not use age-matched normal skin samples. However, we used comparatively young skin samples obtained from subjects whose average age was validated as 22.7 years. As seen in the representative western blots (Figures 6A and B), the protein expression of 14-3-3 epsilon, 14-3-3 tau, and prohibitin in GCMN samples was 1.55-fold, 2-fold, and 2.53-fold, respectively, which was significantly higher than that in normal skin samples. However, the expression of 14-3-3alpha/beta, sigma, and zeta were not significantly different in GCMN and normal skin samples. One possible explanation for the unmatched results between the results of the proteomic and western blot analysis might be the diversity of samples used in the western blot analysis, which unlike the paired samples utilized in the proteomic analysis, were collected from normal skin samples of seven non-GCMN patients.

**Figure 7:**
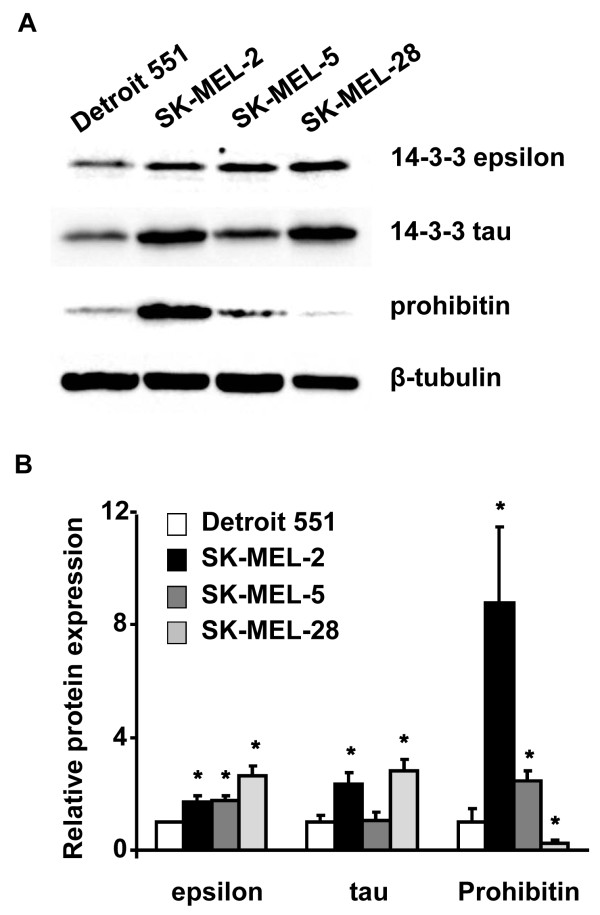
**Validation of protein expression of the 14-3-3 protein family and PHB in normal and melanoma cell lines. A**. Representative western blot images of 14-3-3 epsilon, 14-3-3 tau, and prohibitin in normal skin cells (Detroit 551) and human melanoma cell lines (SK-MEL-2, SK-MEL-5, and SK-MEL-28). **B**. Relative protein expression of 14-3-3 epsilon, 14-3-3 tau, and prohibitin in normal skin cells (Detroit 551) and human melanoma cell lines (SK-MEL-2, SK-MEL-5, and SK-MEL-28) (n = 3 for each cell lines). *p < 0.05, two-tailed unpaired Student’s t-test *vs.* Detroit 551 (normal skin cells).

We further analyzed the expression levels of 14-3-3 epsilon, 14-3-3 tau, and prohibitin in normal skin fibroblast cell line (Detroit 551) and three kinds of melanoma cell lines (SK-MEL-2, SK-MEL-5, and SK-MEL-28) to validate whether our proteomic findings are truly relevant to clinical melanoma. Our results showed that the protein levels of 14-3-3 epsilon were significantly increased in all melanoma cell lines, and the levels of 14-3-3 tau were significantly increased in the SK-MEL-2 and SK-MEL-28 cell lines. Additionally, the protein levels of prohibitin were increased in the SK-MEL-2 and SK-MEL-5 cells, but were decreased in the SK-MEL-28 cell line (Figures 7A and B).

## Discussion

In the present study, the proteomic composition of GCMN was compared with that of normal skin. A major aim of the study was the identification of proteins whose expression is altered in GCMN, which will help understand the altered biological processes in GCMN and help gain an insight into the mechanism of melanotumorigenesis in these malformations. LC-MS/MS analysis showed that 46 of the 438 identified proteins changed in their abundance levels between the normal skin and GCMN samples. In the GCMN samples, 92% of the abundance modified proteins were upregulated, but only 8% were downregulated (Figure 2 and Table 2). The use of different bioinformatic tools showed that GCMN clearly differed from normal skin in terms of protein expression patterns, which suggested that specific biological processes are altered in GCMN. As derived from the GO categories, KEGG pathways, and Reactome_biocarta, these processes were shown to encompass several major biological functions, namely the neurotrophin signaling pathway, downregulated of MTA-3 in ER-negative breast tumors, the cell cycle, phospholipase inhibitor activity, and glycolysis/gluconeogenesis. Strikingly, among these, neurotrophin signaling [17,18], MTA-3 downregulation (Table 3) [19], cell cycle deregulation [20], and glycolysis/gluconeogenesis [21] have been implicated in the development and progression of melanoma and other cancers.

Comparison of systemic properties of the GCMN and metastatic melanoma proteomes revealed that these two different disease proteomes shared at least five proteomic alterations in common and their abundance modified proteins closely interacted with each other (Figure 5A). Because closely related diseases are known to share common proteins or common interactions [22], our results suggested the close relationship between GCMN and melanoma.

Our proteomic analysis also revealed the significantly increased expression of 14 cancer-related proteins in GCMN compared to normal skin samples. Among them, PHB is a molecular maker of malignant cancers, and overexpression of PHB has been reported in melanoma [11,12] and various kinds of cancers, including gastric carcinoma [23], thyroid cancer [24], and hepatocellular carcinoma [25]. This significant upregulation of cancer-related proteins in GCMN, specifically which of melanoma-implicated proteins, strengthened the possible risk of melanotumorigenesis in GCMN.

The 14-3-3 proteins comprise a highly conserved family of proteins whose members are found in both plants and mammals. They mediate signal transduction by binding to phosphoserine-containing proteins and are involved in many biological cellular processes, such as metabolism, protein trafficking, signal transduction, apoptosis, and cell cycle regulation, through interaction with various phosphoserine-containing proteins, such as CDC25 phosphatases, RAF1, and IRS1 proteins. In the present study, 14-3-3 family proteins were estimated to interact with 23 proteins in GCMN and melanoma (Figure 5B), and their average number of interactions was about 2-fold higher than the average number of interactions of other abundance modified proteins. These results suggested that 14-3-3 family proteins could play an important role in the alteration of biological processes in GCMN and melanoma.

The 14-3-3 family proteins consist of seven isoforms: beta, gamma, epsilon, sigma, zeta, tau, and eta. The alpha and sigma isoforms are the phosphoforms of 14-3-3 beta and zeta, respectively. All 14-3-3 proteins are ubiquitously expressed, with the exception of 14-3-3 sigma, which is exclusively expressed in epithelial cells [26]. Among the 14-3-3 family members, the overexpression of the 14-3-3 sigma gene or its respective protein is frequently found in cancers such as ovarian carcinomas [27], pancreatic cancer [28], papillary thyroid carcinoma [29], hepatocellular carcinoma [29], and breast cancer [30]. In our proteomic analysis, the expression of 14-3-3 proteins was significantly higher in GCMN than in normal skin samples, which strongly supports the greater tendency toward melanotumorigenesis in GCMN. In particular, the enhanced expression of 14-3-3 epsilon and tau proteins was clearly shown in western blot analysis (Figure 6).

Compared to the other isoforms, little is known about the molecular and biological role of 14-3-3 tau and epsilon proteins. Like other isoforms, 14-3-3 tau is also involved in cell death and survival processes. For example, 14-3-3 tau binds to ataxia telangiectasia-mutated (ATM)-phosphorylated E2F1 during DNA damage and promotes E2F1 stability, leading to the induction of apoptosis [31], and the deletion of 14-3-3 tau leads to embryonic lethality in a mouse model [32]. Interestingly, a recent study suggested that 14-3-3 tau exhibits an oncogenic role by downregulating p21 in breast cancer [33].

14-3-3 epsilon has been shown to play an essential role in cell development. Studies in *Drosophila* showed that 14-3-3 epsilon is required for the correct timing of mitosis in undisturbed post-blastoderm cell cycle [34]. More recently, defects in neuronal migration during the development of 14-3-3 epsilon-knockout mice were reported [35].

The phosphorylation-induced binding of 14-3-3 epsilon to the pro-apoptotic transcription factor forkhead transcription factor-like 1 (FKHRL1 or FOXO3a) leads to structural changes in 14-3-3 epsilon and inhibits its pro-apoptotic activity [36]. In inflammation and carcinogenesis, 14-3-3 epsilon interacts with key molecules of the mitogen-activated protein kinase signaling module to selectively modulate tumor necrosis factor-alpha-induced nuclear factor-kappa-beta activity [37]. The function and regulatory mechanism of 14-3-3 epsilon in carcinogenesis is controversial and appears to be tumor-specific. Expression of the protein is higher in renal cell carcinoma than that in normal kidney [38]. Moreover, on the basis of their involvement in the tumorigenesis of meningioma, 14-3-3 epsilon, zeta, and theta are thought to be efficient markers for predicting the degree of malignancy of these tumors [39]. In contrast, mRNA and protein expression of 14-3-3 epsilon in laryngeal squamous cell carcinoma tissues was shown to be significantly lower than that in normal tissues [40]. An early role of 14-3-3epsilon in tumorigenesis is suggested by the observation that 14-3-3 epsilon expression is increased in intrinsically aged and photoaged human skin [41]. Interestingly, we found even higher protein levels of 14-3-3 epsilon, 14-3-3 tau, and PHB in GCMN than those in aged skin samples. This result suggested that GCMN may have a higher risk of tumorigenesis than aged skin. Because of the limitation in sample availability, we could not directly determine the expression level of 14-3-3 proteins and PHB in malignant melanoma tissue; however, we demonstrated significantly increased protein expression of 14-3-3 epsilon and tau in two different melanoma cell lines, SK-MEL-2 and SK-MEL-28, compared to normal skin cell line (Detroit 551). This result might support the association of 14-3-3 epsilon and tau upregulation with clinical melanotumorigenesis (Figures 7A and B).

Nevertheless, further studies are needed to validate the functional role of 14-3-3 proteins in melanotumorigenesis through the proteomic comparison of different malignant melanoma patients with giant congenital melanocytic nevi. Furthermore, it is also necessary to carefully validate the biological meaning of the upregulation of melanoma-implicated proteins in GCMN and their role in melanotumorigenesis.

## Conclusion

Taken together, our data suggest that proteomic modifications with tumorigenic potential are present in GCMN, and these proteomic alterations possibly modify six important biological processes or pathways that include melanosome, neurotrophin signaling pathway, downregulated of MTA-3 in ER-negative breast tumors, cell cycle, phospholipase inhibitor activity, and glycolysis/gluconeogenesis These pathways may be significantly altered in GCMN skins. The intensive alteration of 14-3-3 family proteins and PHB possibly acts as a central regulator of GCMN biological pathway remodeling, which may have an important role in the development of GCMN and could be associated with melanotumorigenesis.

## Materials and methods

### Patients

A total of 10 normal and GCMN skin samples, which were defatted, were obtained from patients who underwent excision procedures at the Department of Plastic Surgery, Inje University Ilsan Paik Hospital, Korea. The collection and use of the samples were approved by the Institutional Review Board of Inje University Ilsan Paik Hospital (IRB No. IB-0902-015). The present study was carried out in accordance with *The Code of Ethics of the World Medical Association (Declaration of Helsinki) for experiments involving humans*.

### Cell lines and culture conditions

The human embryo skin cell line Detroit 551 and the human malignant melanoma cell lines SK-MEL-2, SK-MEL-5, and SK-MEL-28 were obtained from the American Type Culture Collection (ATCC; Rockville, MD). The culture medium used throughout these experiments was RPMI-1640 (Lonza, Verviers, Belgium) containing 10% fetal bovine serum (PAA, Pasching, Austria) and 100 μg/ml penicillin-streptomycin (Lonza). The cells were incubated at 37°C in a humidified atmosphere of 5% CO_2_.

### Sample preparation for proteomics

Three paired normal and GCMN skin samples were selected for 1D-LC-MS/MS proteomic analysis to exclude environmental bias. Excised skin samples were ground to a powder in liquid nitrogen, dissolved in lysis buffer (9 M urea, 2 M thiourea, 4% CHAPS (3-[(3-cholamidopropyl)dimethylammonio]-1-propanesulfonate), 40 mM dithiothreitol (DTT), and 1% protease inhibitor cocktail), vortexed, and incubated on ice for 1 h. The mixture was then centrifuged (10,000 × *g*, 30 min, 4°C), and the total proteins contained in the supernatant were used for the experiments. The total protein content of the solution was determined using the 2D Quant kit (GE Healthcare, Milwaukee, WI), with bovine serum albumin (0–50 mg/ml) as the standard.

### 1D-LC-MS/MS

Protein separation and LC-MS analysis were performed as previously described [42]. Briefly, dissolved skin proteins were separated on a 12% polyacrylamide gel by SDS-PAGE. The gels were washed three times with ddH_2_O for 5 min each and stained with Bio-Safe Coomassie stain solution (Coomassie G250 stain; Bio-Rad, Hercules, CA) for 1 h, with gentle shaking at room temperature. The Coomassie-stained gels were evenly sliced into 15 slices and then destained by incubation in 75 mM ammonium bicarbonate/40% ethanol (1:1). Disulfides were reduced by treatment with 5 mM DTT/25 mM ammonium bicarbonate at 60°C for 30 min, followed by alkylation with 55 mM iodoacetoamide at room temperature for 30 min. The gel pieces were then dehydrated in 100% acetonitrile (ACN), dried, and swollen overnight at 37°C in 10 μl 25 mM ammonium bicarbonate buffer containing 20 μg modified sequencing-grade trypsin (Roche Applied Science, Indianapolis, IN) per ml. The tryptic peptide mixture was eluted from the gel using 0.1% formic acid. LC-MS/MS analysis was performed using a ThermoFinnigan ProteomeX workstation LTQ linear ion trap MS (Thermo Electron, San Jose, CA) equipped with a nanospray ionization (NSI) source (Thermo Electron). Briefly, 12 μl peptide sample obtained from the in-gel digestion was injected and loaded onto a peptide trap cartridge (Agilent, Palo Alto, CA). Trapped peptides were eluted onto a 10-cm reversed-phase PicoFrit column packed in-house with 5-μm, 300-Å pore size C18 and separated by gradient elution. The mobile phases consisted of H_2_O and ACN, both containing 0.1% v/v formic acid. The flow rate was maintained at 200 nl/min. The gradient started at 2% ACN, then reached 60% ACN in 50 min, 80% ACN in the next 5 min, and 100% H_2_O in the final 15 min. Data-dependent acquisition (*m/z* 400–1800) was enabled, and each MS survey scan was followed by five MS/MS scans within 30 s, with the dynamic exclusion option enabled. The spray voltage was 1.9 kV, the temperature of the ion transfer tube was 195°C, and the normalized collision energy was 35% [42].

Data-analyzed tandem mass spectra were extracted, and the charge state was deconvoluted and deisotoped using the Sorcerer 3.4 beta2 platform (Sorcerer software 3.1.4, Sorcerer Web interface 2.2.0 r334, and Trans-, Proteomic Pipeline 2.9.5). All MS/MS samples were analyzed using SEQUEST (version v.27, rev. 11; ThermoFinnigan, San Jose, CA), which was set to search the ipiHuman 3.29 database (IPI ver.3.29, 40131 entries), with semitrypsin as the digestion enzyme. The search used a fragment-ion mass tolerance of 1.00 Da and a parent-ion mass tolerance of 1.5 Da. Iodoacetamide-derivatized cysteine was specified as a fixed modification. Methionine oxidation, iodoacetamide derivatizion of cysteine, and phosphorylation of serine, threonine, and tyrosine were specified as variable modifications. The Scaffold software (version Scaffold-2.0; Proteome Software Inc., Portland, OR) was used to validate MS/MS-based peptide and protein identifications. Peptide identifications were accepted if their probability was >95.0%, as specified by the Peptide Prophet algorithm, and if they contained at least one identified peptide. Protein probabilities were assigned by the Protein Prophet algorithm. Proteins containing similar peptides such that they could not be differentiated based on MS/MS analysis alone were grouped to satisfy the principles of parsimony. After identifying the proteins, each dataset was used for a subtractive analysis by semi-quantitative normalized spectral counts, which were normalized by total spectral counts in the Scaffold program [43].

### Bioinformatics analysis

A systemic bioinformatics analysis of the GCMN proteome was conducted using the Search Tool for the Retrieval of Interacting Genes/Proteins (STRING 8.3) [44], the Protein Analysis Through Evolutionary Relationships classification system (PANTHER 7.0) [45], the National Center for Biotechnology Information (NCBI) COG database [46], Cytoscape, and ClueGO [47].

### Western blot analysis

Protein expressions of 14-3-3 alpha + beta (30 kDa), 14-3-3 epsilon (29 kDa), 14-3-3 zeta (28 kDa), 14-3-3 sigma (25 kDa), 14-3-3 tau (31 kDa), and prohibitin (30 kDa) in normal (n = 7) and GCMN (n = 7) samples were analyzed by western blots to confirm the proteomic results. In addition, protein expressions of 14-3-3 epsilon, 14-3-3 tau, and prohibitin in a normal cell line (Detroit 551) and melanoma cell lines (SK-MEL-2, SK-MEL-5, and SK-MEL-28) were analyzed by western blots. Relative expression of each protein was normalized to an internal standard protein, β-tubulin (55 kDa) or glyceraldehyde-3-phosphate dehydrogenase (37 kDa) (Abcam, Cambridge, MA). The values were expressed as mean ± standard error.

### Statistical analysis

Two-tailed unpaired Student’s t-test in the Scaffold (version 2.06.02) software was used to compare abundance of each protein in the normal and GCMN skin samples. Using the Origin software (version 7.0220, OriginLabs, MA, USA), Bonferroni correction was applied to control the rate of false positives in the comparison of means of each protein’s abundance. A two-tailed unpaired Student’s t-test was used to compare the results of western blot analysis between the normal and GCMN skin samples. p < 0.05 was considered statistically significant.

## Abbreviations

GCMN: Giant congenital melanocytic nevus; CTSD: cathepsin D; PDIA3: Protein disulfide-isomerase A3 precursor; PHB: Prohibitin; HSPA8: Heat shock protein 70; PHGDH: D-3-phosphoglycerate dehydrogenase; ATP5B: ATP synthase subunit beta; KRT1: Cytokeratin-1; FLG2: Filaggrin; HRNR: Hornerin; ADH1B: Alcohol dehydrogenase 1B; CSPG4: Chondroitin sulfate proteoglycan 4; PEBP1: Phosphatidylethanolamine-binding protein; RPSA: Ribosomal protein SA; COG: Clusters of Orthologous Groups; STRING: Search Tool for the Retrieval of Interacting Genes/Proteins; PANTHER: Protein Analysis Through Evolutionary Relationships; KEGG: Kyoto Encyclopedia of Genes and Genomes; GO: Gene Ontology.

## Competing interests

The authors declare that they have no competing interests.

## Author’s contributions

HKK performed the proteomic analysis including its design, coordination, analysis of the data, and drafted the manuscript. YKK provided GCMN and normal skin samples and was largely involved in the design of the study and in writing the manuscript. ISS, SRL, SHJ, MHK, and DYS performed sample preparation and western blot analysis. NK, BDR, KSK, KCT, and CGP critically revised the clinical aspect of the manuscript. JYC carried out the MS/MS proteomic analysis. JH conceived the overall experimental design and manuscript preparation. All authors read and approved the final manuscript.

## Supplementary Material

Additional file 1 **Figure S1.** Peptide mass peak of 15 slices dissected from 1D gels of normal skin and GCMN. Click here for file

Additional file 2 **Table S1.** Identified proteins in skin samples of Normal and GCMN patients. Click here for file

Additional file 3 **Figure S2.** The functional network and enriched functional group of GCMN was analyzed using ClueGO, a biological term enrichment analyzer. The following proteins were significantly enriched in GCMN: melanosome (GO_cellular component), neurotrophin signaling pathway (KEGG pathway), downregulated of MTA-3 in ER-negative breast tumors (Biocarta), cell cycle (KEGG pathway), phospholipase inhibitor activity (GO_molecular function), and glycolysis/gluconeogenesis (KEGG pathway). Click here for file

Additional file 4 **Table S2.** Integrated densitometry value of Western blot band. Click here for file

Additional file 5 **Table S3.** Statistical analysis result of western blot. Click here for file
