# Supplementary material for: Human giant congenital melanocytic nevus exhibits potential proteomic alterations leading to melanotumorigenesis
Source: Proteome Sci. 2012 Aug 20;10:50. doi: 10.1186/1477-5956-10-50 (PMC3575290; doi:10.1186/1477-5956-10-50)
Supplement: Additional file 1 — Figure S1. Peptide mass peak of 15 slices dissected from 1D gels of normal skin and GCMN. [file 1477-5956-10-50-S1.pdf]

Supplementary Figures

Supplementary Figure 1

Normal 1

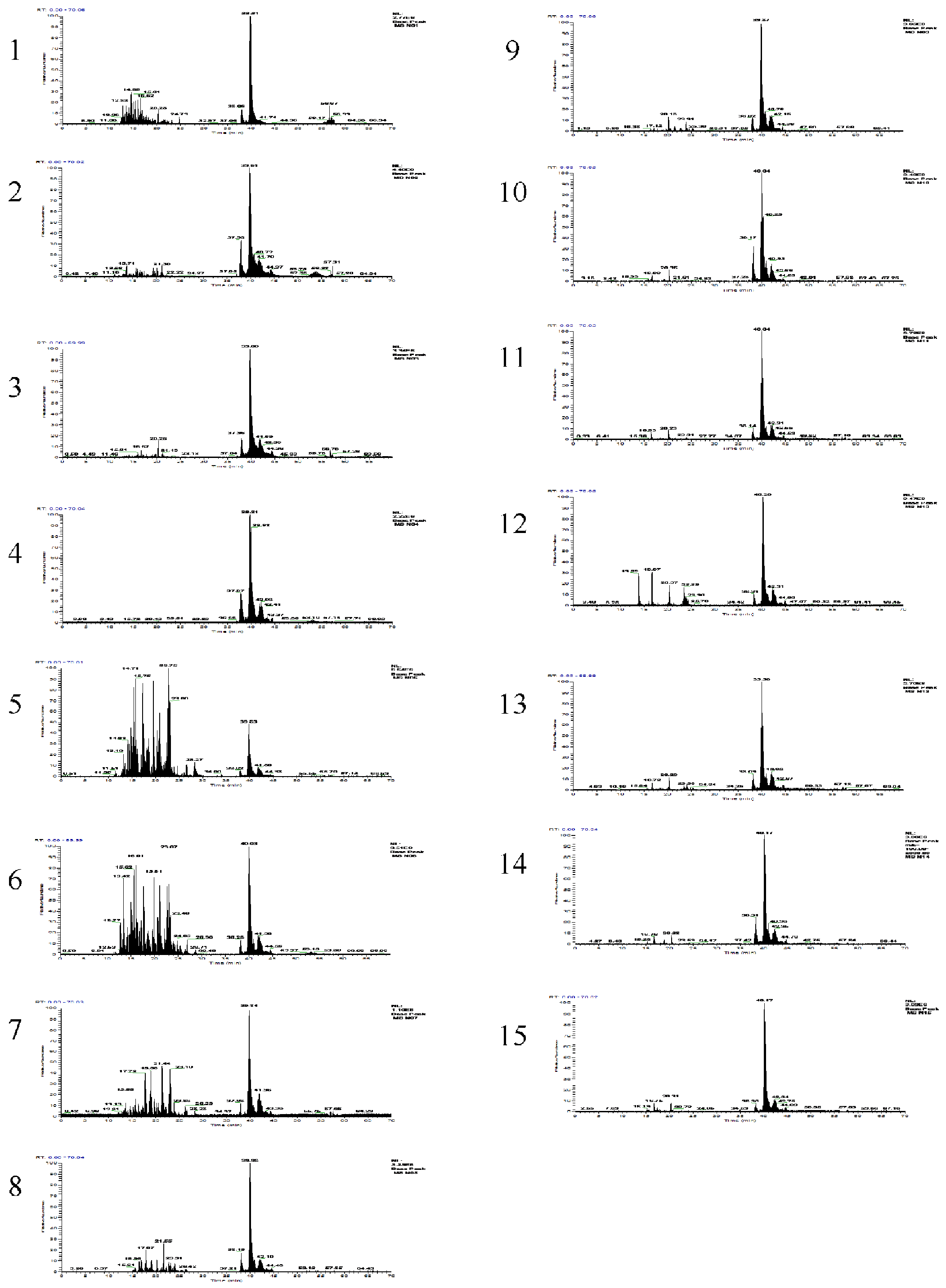

Supplementary Figure 1

Normal 2

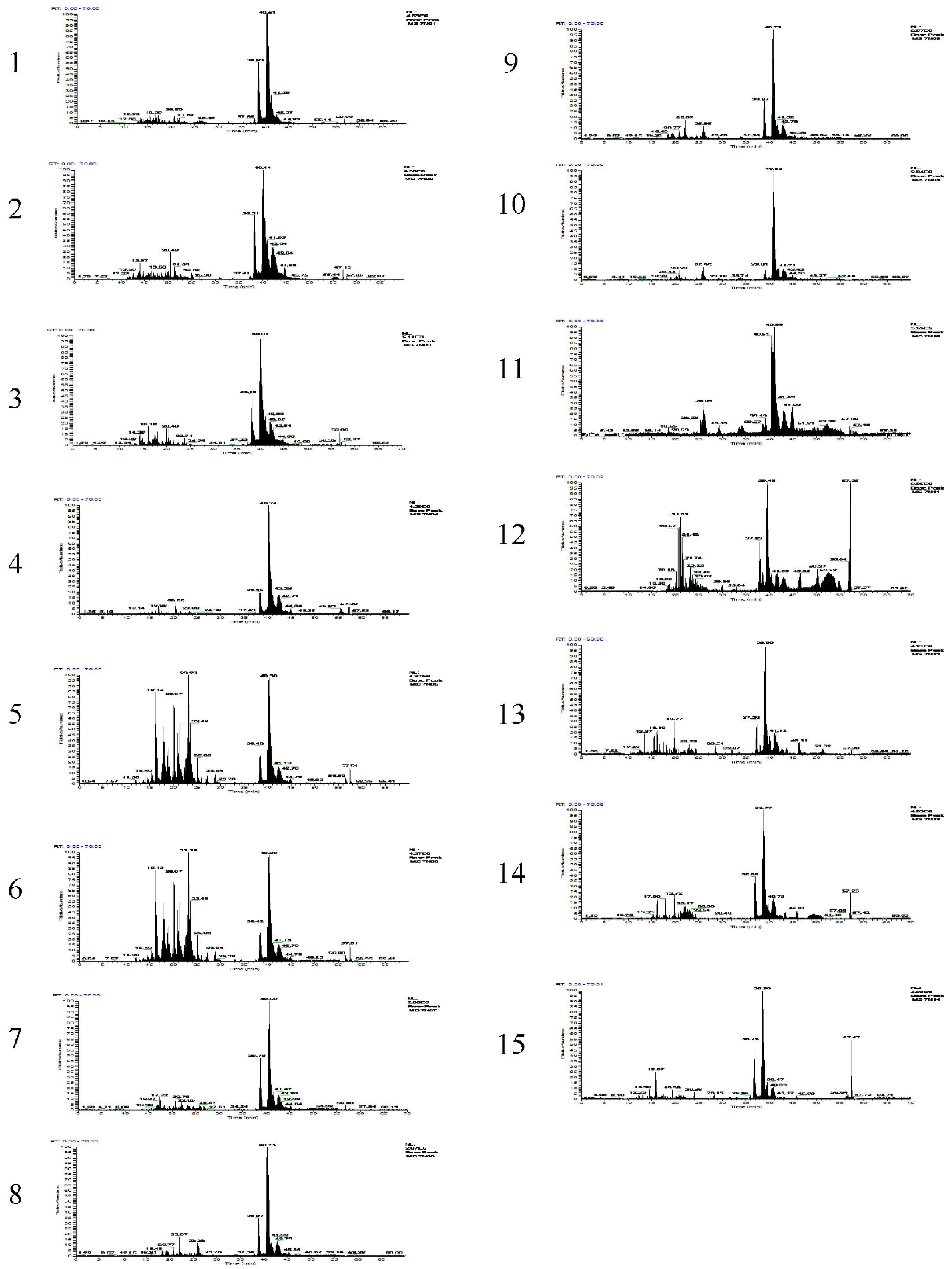

Supplementary Figure 1

Normal 3

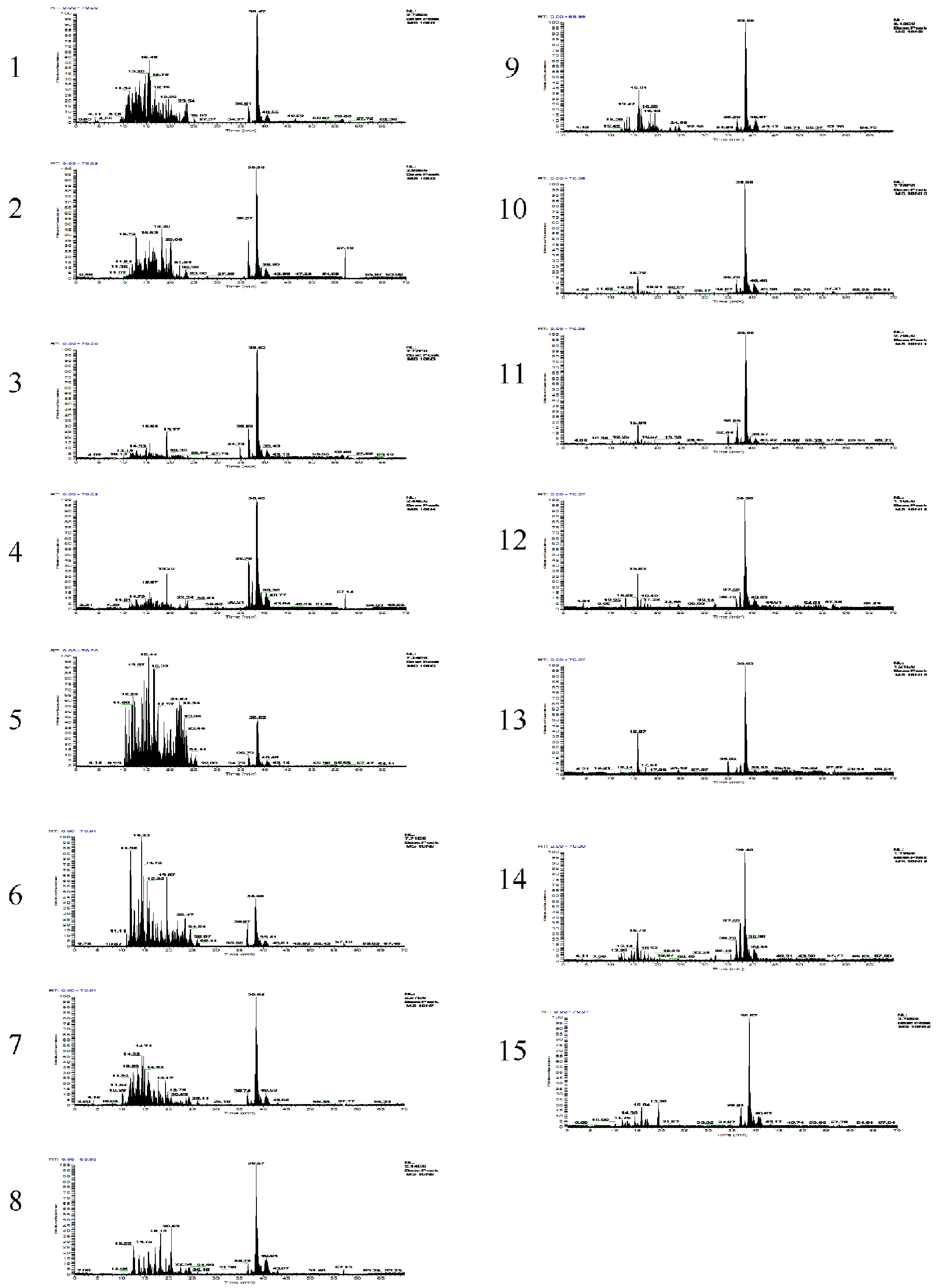

Supplementary Figure 1

CMN 1

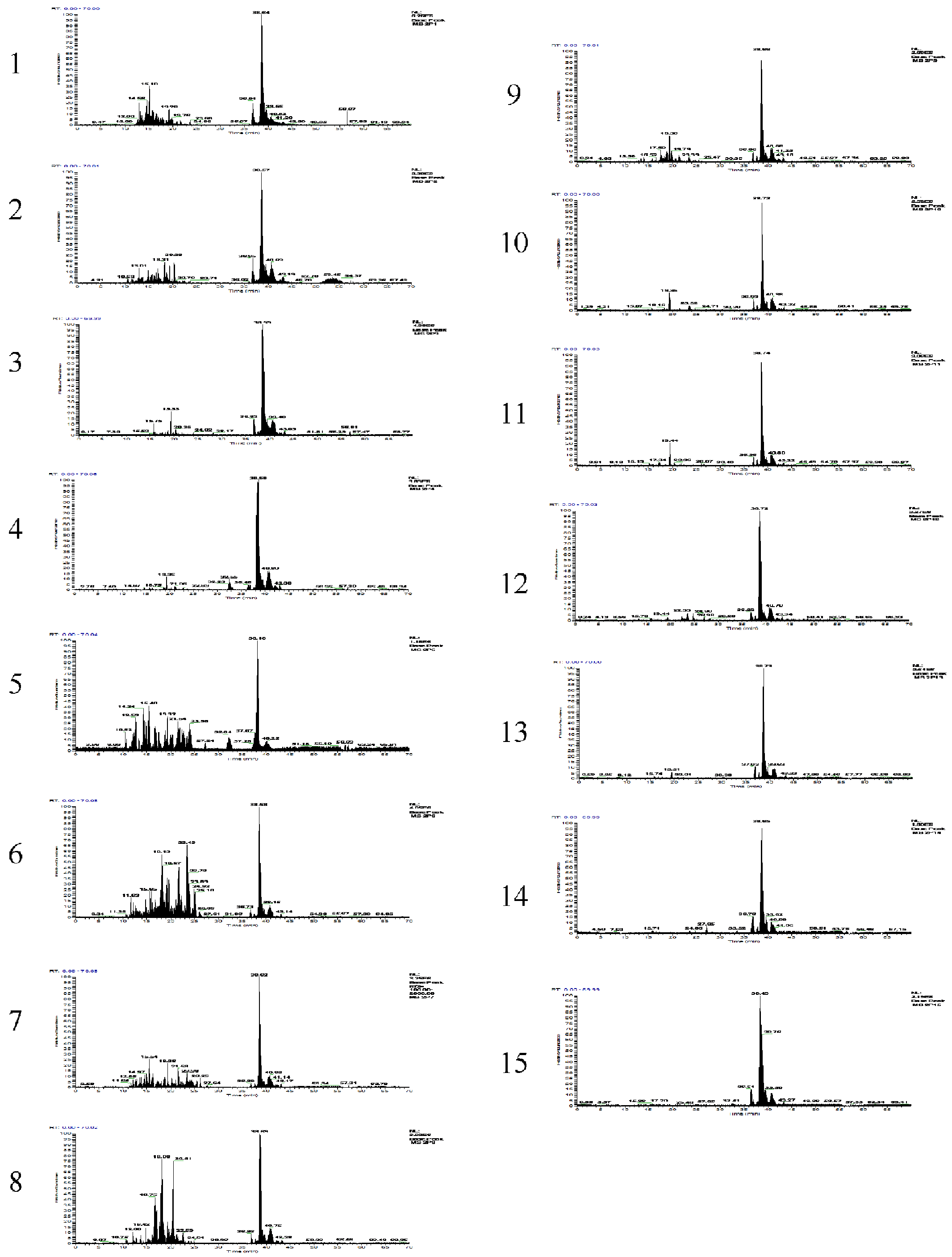

Supplementary Figure 1

CMN 2

1
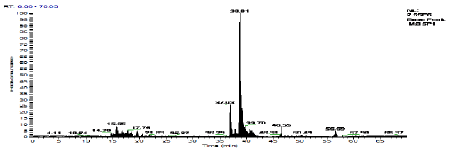

2
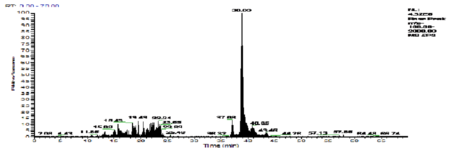

3
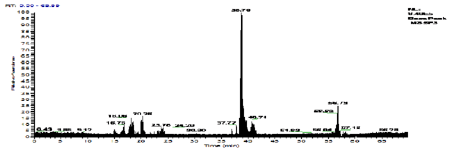

4
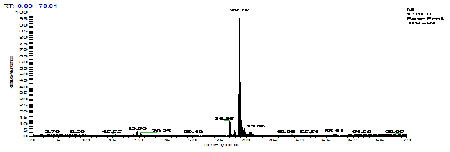

5
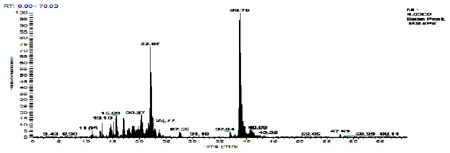

6
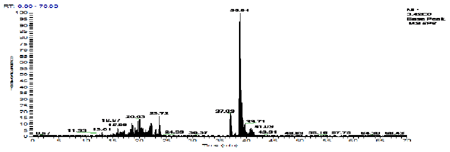

7
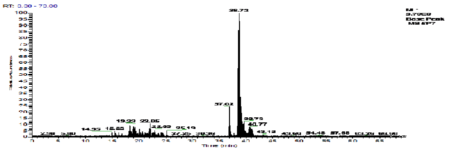

8
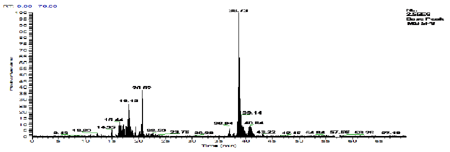

9
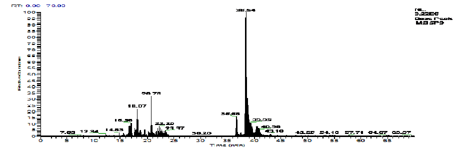

10
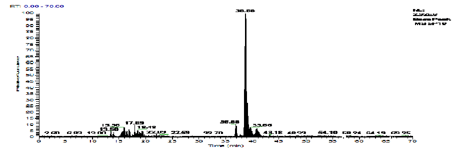

11
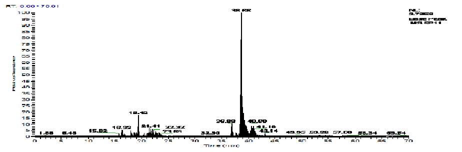

12
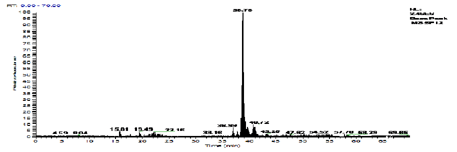

13
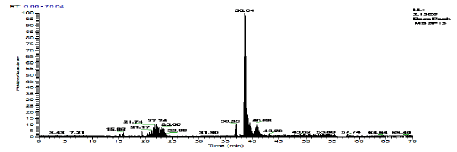

14
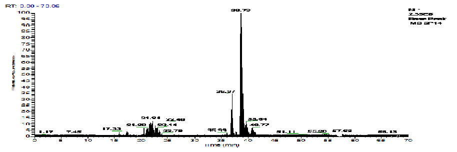

15
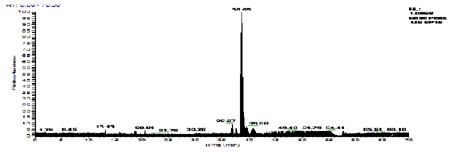

Supplementary Figure 1

CMN 3

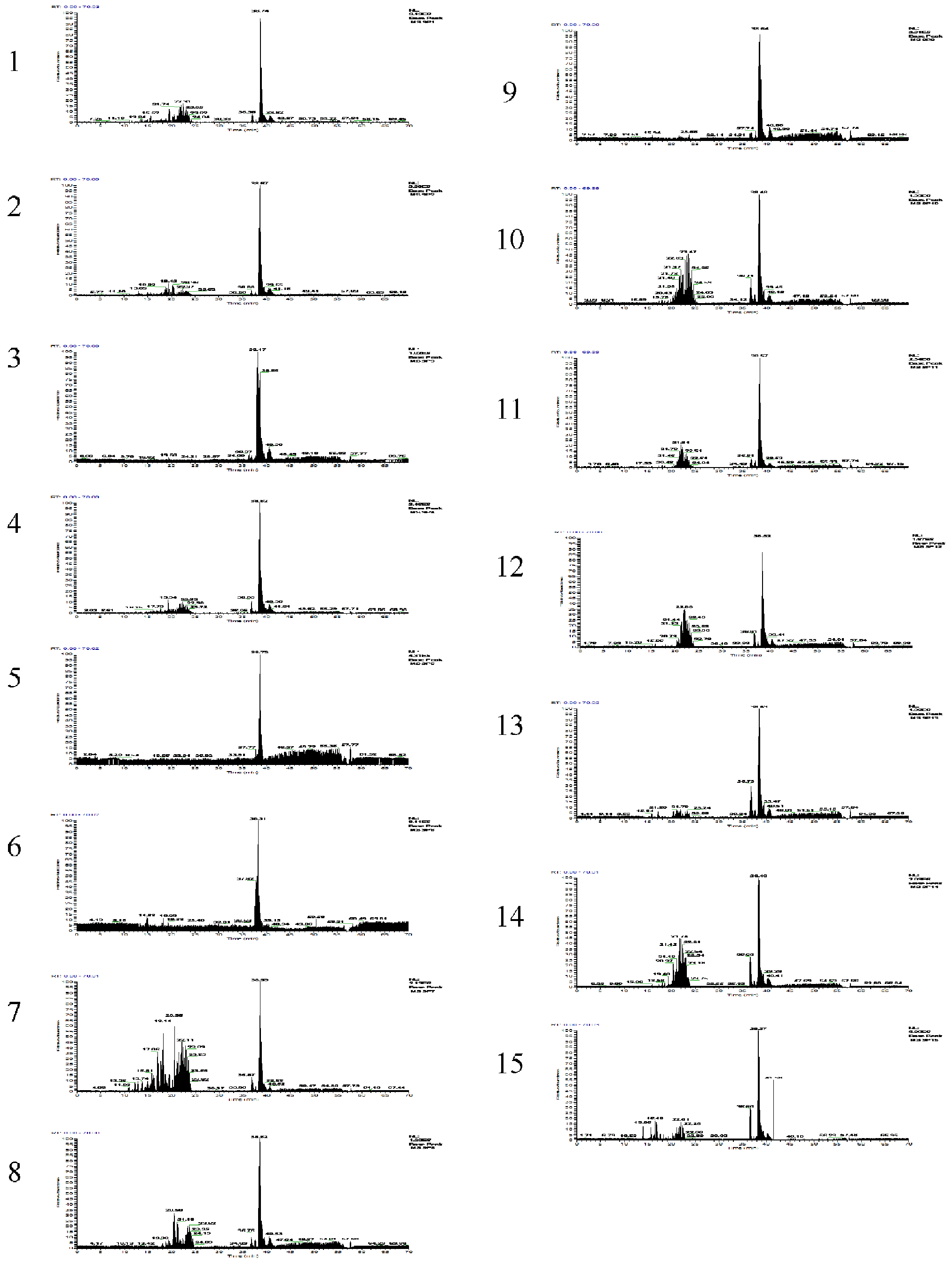

Figure S1. Peptide mass peak of 15 bands dissected from 1D gels of normal skin and GCMN.
