# Supplementary material for: Human giant congenital melanocytic nevus exhibits potential proteomic alterations leading to melanotumorigenesis
Source: Proteome Sci. 2012 Aug 20;10:50. doi: 10.1186/1477-5956-10-50 (PMC3575290; doi:10.1186/1477-5956-10-50)
Supplement: Additional file 2 — Table S1. Identified proteins in skin samples of Normal and GCMN patients. [file 1477-5956-10-50-S2.doc]

**Supplementary Table S1**

Table S1. Identified proteins in skin samples of Normal and GCMN patients

| # | Identified Proteins (438) | Accession  Number^1^ | MW | T-Test^2^ | Normalized count number of unique peptide | | | | | |
| --- | --- | --- | --- | --- | --- | --- | --- | --- | --- | --- |
|  |  |  |  |  | CMN 1 | CMN 2 | CMN 3 | Nor 1 | Nor 2 | Nor 3 |
| 1 | ALB Isoform 1 of Serum albumin | IPI00745872 | 69 kDa | 0.14 | 726.97 | 1,072.75 | 478.14 | 2,189.40 | 1,338.76 | 973.9 |
| 2 | KRT10 Keratin, type I cytoskeletal 10 | IPI00009865 | 60 kDa | 0.57 | 606.48 | 288.17 | 515.34 | 357.83 | 462.12 | 1,001.57 |
| 3 | KRT1 Keratin, type II cytoskeletal 1 | IPI00220327 | 66 kDa | 0.016 | 464.9 | 491.91 | 536.01 | 605.44 | 720.14 | 747.82 |
| 4 | COL6A3 alpha 3 type VI collagen isoform 1 precursor | IPI00022200 | 344 kDa | 0.29 | 460.88 | 395.1 | 417.51 | 340.88 | 439.91 | 362.05 |
| 5 | KRT2 Keratin, type II cytoskeletal 2 epidermal | IPI00021304 | 66 kDa | 0.28 | 212.87 | 160.97 | 184.64 | 254.38 | 230.53 | 932 |
| 6 | ACTB Actin, cytoplasmic 1 | IPI00021439 | 42 kDa | 0.0027 | 187.77 | 251.02 | 214.96 | 100.06 | 94.11 | 86.96 |
| 7 | COL6A1 Collagen alpha-1(VI) chain | IPI00291136 | 109 kDa | 0.45 | 123.5 | 177.85 | 159.84 | 111.08 | 169.2 | 120.95 |
| 8 | KRT5 Keratin, type II cytoskeletal 5 | IPI00009867 | 62 kDa | 0.90 | 346.42 | 201.49 | 231.49 | 156.87 | 234.76 | 357.31 |
| 9 | KRT14 Keratin, type I cytoskeletal 14 | IPI00384444 | 52 kDa | 0.38 | 278.14 | 133.95 | 321.06 | 89.88 | 137.47 | 274.3 |
| 10 | IGHG1 IGHG1 protein | IPI00448925 | 60 kDa | 0.79 | 98.4 | 119.32 | 86.81 | 170.44 | 102.57 | 60.08 |
| 11 | KRT9 Keratin, type I cytoskeletal 9 | IPI00019359 | 62 kDa | 0.078 | 71.29 | 108.06 | 79.92 | 139.06 | 123.72 | 103.56 |
| 12 | ANXA2 annexin A2 isoform 1 | IPI00418169 | 40 kDa | 0.0059 | 123.5 | 123.82 | 143.3 | 62.75 | 89.89 | 67.19 |
| 13 | FGG Isoform Gamma-A of Fibrinogen gamma chain | IPI00219713 | 49 kDa | 0.35 | 3.01 | 27.02 | 30.31 | 51.72 | 309.84 | 1 |
| 14 | DSP Isoform DPI of Desmoplakin | IPI00013933 | 332 kDa | 0.30 | 117.48 | 27.02 | 117.12 | 30.53 | 69.79 | 47.43 |
| 15 | COL6A2 Isoform 2C2 of Collagen alpha-2(VI) chain | IPI00304840 | 109 kDa | 0.85 | 64.26 | 61.91 | 68.9 | 48.33 | 78.25 | 63.24 |
| 16 | TF Serotransferrin | IPI00022463 | 77 kDa | 0.60 | 61.25 | 42.77 | 74.41 | 84.79 | 72.97 | 45.85 |
| 17 | LMNA Isoform A of Lamin-A/C | IPI00021405 | 74 kDa | 0.10 | 51.21 | 96.81 | 148.82 | 1 | 58.16 | 31.62 |
| 18 | FGB Fibrinogen beta chain | IPI00298497 | 56 kDa | 0.32 | 1 | 38.27 | 6.89 | 50.88 | 225.24 | 2.37 |
| 19 | COL14A1 Isoform 1 of Collagen alpha-1(XIV) chain | IPI00176193 | 194 kDa | 0.52 | 37.15 | 31.52 | 4.13 | 183.16 | 12.69 | 2.37 |
| 20 | A2M Alpha-2-macroglobulin | IPI00478003 | 163 kDa | 0.20 | 16.07 | 10.13 | 8.27 | 151.78 | 70.85 | 5.53 |
| 21 | KRT72 Isoform 1 of Keratin, type II cytoskeletal 72 | IPI00103481 | 56 kDa | 0.63 | 61.25 | 50.65 | 84.05 | 33.92 | 37.01 | 222.92 |
| 22 | IGKV1-5 IGKV1-5 protein | IPI00430820 | 26 kDa | 0.58 | 22.09 | 39.4 | 45.47 | 98.36 | 35.95 | 18.18 |
| 23 | TUBB Tubulin beta chain | IPI00011654 | 50 kDa | 0.098 | 57.23 | 135.08 | 38.58 | 5.94 | 16.92 | 15.81 |
| 24 | KRT86 Keratin type II cuticular Hb6 | IPI00182655 | 53 kDa | 0.33 | 193.79 | 11.26 | 13.78 | 1 | 10.57 | 6.32 |
| 25 | GAPDH Glyceraldehyde-3-phosphate dehydrogenase | IPI00219018 | 36 kDa | 0.035 | 39.16 | 65.29 | 44.09 | 19.5 | 28.55 | 19.76 |
| 26 | POSTN Periostin, osteoblast specific factor | IPI00410241 | 90 kDa | 0.24 | 25.1 | 43.9 | 92.32 | 4.24 | 41.24 | 21.34 |
| 27 | ENO1 Isoform alpha-enolase of Alpha-enolase | IPI00465248 | 47 kDa | 0.080 | 27.11 | 93.43 | 57.87 | 11.02 | 19.03 | 13.44 |
| 28 | FGA Isoform 1 of Fibrinogen alpha chain | IPI00021885 | 95 kDa | 0.35 | 1 | 5.63 | 8.27 | 16.11 | 173.43 | 1.58 |
| 29 | JUP Junction plakoglobin | IPI00554711 | 82 kDa | 0.30 | 55.23 | 15.76 | 68.9 | 1 | 33.84 | 35.57 |
| 30 | ANXA5 Annexin A5 | IPI00329801 | 36 kDa | 0.025 | 32.13 | 59.66 | 50.98 | 21.2 | 1 | 15.02 |
| 31 | KRT6A Keratin, type II cytoskeletal 6A | IPI00300725 | 60 kDa | 0.95 | 213.87 | 126.07 | 115.75 | 117.86 | 144.87 | 184.98 |
| 32 | YWHAZ 14-3-3 protein zeta/delta | IPI00021263 | 28 kDa | 0.0021 | 39.16 | 29.27 | 33.07 | 12.72 | 11.63 | 14.23 |
| 33 | ACTN4 Alpha-actinin-4 | IPI00013808 | 105 kDa | 0.34 | 45.18 | 7.88 | 38.58 | 16.11 | 24.32 | 12.65 |
| 34 | PRDX1 Peroxiredoxin-1 | IPI00000874 | 22 kDa | 0.50 | 20.08 | 21.39 | 38.58 | 13.57 | 31.72 | 16.6 |
| 35 | ALB Uncharacterized protein ALB | IPI00022434 | 72 kDa | 0.14 | 723.96 | 1,084.00 | 482.27 | 2,173.29 | 1,341.93 | 970.74 |
| 36 | COL1A1 Collagen alpha-1(I) chain | IPI00297646 | 139 kDa | 0.96 | 33.14 | 14.63 | 15.16 | 30.53 | 20.09 | 13.44 |
| 37 | TUBA1A Tubulin alpha-1A chain | IPI00180675 | 50 kDa | 0.24 | 23.09 | 67.54 | 13.78 | 9.33 | 15.86 | 9.49 |
| 38 | TPI1 Isoform 1 of Triosephosphate isomerase | IPI00465028 | 31 kDa | 0.15 | 22.09 | 28.14 | 30.31 | 16.11 | 26.44 | 13.44 |
| 39 | LUM Lumican | IPI00020986 | 38 kDa | 0.71 | 19.08 | 12.38 | 37.2 | 6.78 | 17.98 | 31.62 |
| 40 | IGLV3-25 IGLV3-25 protein | IPI00550162 | 25 kDa | 0.21 | 17.07 | 11.26 | 15.16 | 39.85 | 28.55 | 11.86 |
| 41 | ANXA1 Annexin A1 | IPI00218918 | 39 kDa | 0.030 | 22.09 | 42.77 | 35.83 | 16.11 | 5.29 | 11.86 |
| 42 | DCN Isoform A of Decorin | IPI00012119 | 40 kDa | 0.47 | 37.15 | 9.01 | 23.42 | 16.11 | 10.57 | 22.13 |
| 43 | EEF1A1 Elongation factor 1-alpha 1 | IPI00396485 | 50 kDa | 0.012 | 24.1 | 28.14 | 35.83 | 9.33 | 9.52 | 15.81 |
| 44 | LDHA Isoform 1 of L-lactate dehydrogenase A chain | IPI00217966 | 37 kDa | 0.62 | 21.09 | 27.02 | 12.4 | 11.02 | 24.32 | 15.81 |
| 45 | VIM Vimentin | IPI00418471 | 54 kDa | 0.17 | 8.03 | 88.93 | 28.94 | 1 | 3.17 | 1 |
| 46 | C3 Complement C3 (Fragment) | IPI00783987 | 187 kDa | 0.31 | 2.01 | 1 | 9.65 | 71.23 | 15.86 | 1 |
| 47 | HSPA8 Isoform 1 of Heat shock cognate 71 kDa protein | IPI00003865 | 71 kDa | 0.0016 | 25.1 | 34.9 | 33.07 | 3.39 | 7.4 | 7.11 |
| 48 | CA1 Carbonic anhydrase 1 | IPI00215983 | 29 kDa | 0.85 | 15.06 | 14.63 | 20.67 | 19.5 | 24.32 | 9.49 |
| 49 | PPIA Peptidyl-prolyl cis-trans isomerase A | IPI00419585 | 18 kDa | 0.12 | 21.09 | 16.88 | 24.8 | 10.18 | 19.03 | 12.65 |
| 50 | HBB Hemoglobin subunit beta | IPI00654755 | 16 kDa | 0.30 | 5.02 | 6.75 | 15.16 | 18.65 | 40.18 | 5.53 |
| 51 | GSN Isoform 1 of Gelsolin | IPI00026314 | 86 kDa | 0.97 | 18.07 | 10.13 | 17.91 | 5.09 | 24.32 | 17.39 |
| 52 | RAB10 Ras-related protein Rab-10 | IPI00016513 | 23 kDa | 0.87 | 10.04 | 19.14 | 26.18 | 19.5 | 27.49 | 3.95 |
| 53 | KRT31 Keratin, type I cuticular Ha1 | IPI00032513 | 47 kDa | 0.24 | 93.38 | 23.64 | 39.96 | 1 | 25.38 | 32.41 |
| 54 | ACTA2 Actin, aortic smooth muscle | IPI00008603 | 42 kDa | 0.070 | 82.34 | 146.33 | 99.21 | 66.99 | 64.51 | 49.01 |
| 55 | LEMD3 Inner nuclear membrane protein Man1 | IPI00032491 | 100 kDa | 0.56 | 5.02 | 7.88 | 15.16 | 13.57 | 4.23 | 1 |
| 56 | PKM2 Isoform M1 of Pyruvate kinase isozymes M1/M2 | IPI00220644 | 58 kDa | 0.53 | 32.13 | 9.01 | 6.89 | 3.39 | 10.57 | 15.81 |
| 57 | SFN Isoform 1 of 14-3-3 protein sigma | IPI00013890 | 28 kDa | 0.027 | 32.13 | 22.51 | 31.69 | 16.11 | 13.75 | 19.76 |
| 58 | ALDOA Fructose-bisphosphate aldolase A | IPI00465439 | 39 kDa | 0.034 | 13.05 | 29.27 | 26.18 | 1.7 | 8.46 | 7.11 |
| 59 | HSP90AA1 heat shock protein 90kDa alpha (cytosolic), class A member 1 isoform 1 | IPI00382470 | 98 kDa | 0.020 | 21.09 | 24.76 | 13.78 | 3.39 | 9.52 | 4.74 |
| 60 | TPSB2 TPSB2 protein | IPI00419942 | 31 kDa | 0.043 | 21.09 | 14.63 | 13.78 | 10.18 | 3.17 | 8.7 |
| 61 | PGK1 Phosphoglycerate kinase 1 | IPI00169383 | 45 kDa | 0.10 | 15.06 | 29.27 | 11.02 | 10.18 | 4.23 | 3.95 |
| 62 | ATP5B ATP synthase subunit beta, mitochondrial | IPI00303476 | 57 kDa | 0.032 | 28.11 | 21.39 | 13.78 | 1 | 10.57 | 1 |
| 63 | KRT16 Keratin, type I cytoskeletal 16 | IPI00217963 | 51 kDa | 0.67 | 169.69 | 96.81 | 213.58 | 65.29 | 106.8 | 226.87 |
| 64 | SERPINA1 Isoform 1 of Alpha-1-antitrypsin | IPI00553177 | 47 kDa | 0.66 | 9.04 | 15.76 | 1.38 | 22.05 | 11.63 | 2.37 |
| 65 | HSPA1A;HSPA1B Heat shock 70 kDa protein 1 | IPI00304925 | 70 kDa | 0.031 | 21.09 | 28.14 | 19.29 | 4.24 | 14.8 | 9.49 |
| 66 | PLEC1 Isoform 1 of Plectin-1 | IPI00014898 | 532 kDa | 0.32 | 11.05 | 24.76 | 12.4 | 3.39 | 19.03 | 1.58 |
| 67 | KRT85 Keratin type II cuticular Hb5 | IPI00032541 | 56 kDa | 0.31 | 155.64 | 14.63 | 13.78 | 1 | 1 | 7.11 |
| 68 | PRDX2 Peroxiredoxin-2 | IPI00027350 | 22 kDa | 0.67 | 13.05 | 10.13 | 22.05 | 11.87 | 31.72 | 11.86 |
| 69 | MYH9 Isoform 1 of Myosin-9 | IPI00019502 | 227 kDa | 0.14 | 15.06 | 31.52 | 6.89 | 1.7 | 8.46 | 1.58 |
| 70 | - Uncharacterized protein ENSP00000348237 | IPI00453476 | 29 kDa | 0.0012 | 16.07 | 16.88 | 17.91 | 7.63 | 3.17 | 5.53 |
| 71 | MDH2 Malate dehydrogenase, mitochondrial | IPI00291006 | 36 kDa | 0.076 | 18.07 | 14.63 | 9.65 | 4.24 | 9.52 | 7.91 |
| 72 | PPIB peptidylprolyl isomerase B precursor | IPI00646304 | 24 kDa | 0.75 | 7.03 | 7.88 | 17.91 | 11.87 | 10.57 | 6.32 |
| 73 | LGALS7;LGALS7B Galectin-7 | IPI00219221 | 15 kDa | 0.57 | 6.02 | 6.75 | 12.4 | 5.09 | 19.03 | 9.49 |
| 74 | LMNB1 Lamin-B1 | IPI00217975 | 66 kDa | 0.28 | 13.05 | 10.13 | 4.13 | 8.48 | 4.23 | 3.16 |
| 75 | COL12A1 Isoform 4 of Collagen alpha-1(XII) chain | IPI00302944 | 325 kDa | 0.73 | 18.07 | 2.25 | 1 | 25.44 | 2.11 | 3.95 |
| 76 | KRT17 Keratin, type I cytoskeletal 17 | IPI00450768 | 48 kDa | 0.61 | 100.41 | 76.54 | 139.17 | 1 | 59.22 | 1 |
| 77 | LOC100133739 Putative uncharacterized protein DKFZp686C15213 | IPI00426051 | 51 kDa | 0.28 | 24.1 | 25.89 | 17.91 | 39.85 | 25.38 | 23.72 |
| 78 | CLTC Isoform 1 of Clathrin heavy chain 1 | IPI00024067 | 192 kDa | 0.36 | 12.05 | 16.88 | 5.51 | 11.02 | 8.46 | 1.58 |
| 79 | LDHB L-lactate dehydrogenase B chain | IPI00219217 | 37 kDa | 0.11 | 18.07 | 29.27 | 9.65 | 6.78 | 6.34 | 8.7 |
| 80 | VAT1 Synaptic vesicle membrane protein VAT-1 homolog | IPI00156689 | 42 kDa | 0.17 | 2.01 | 31.52 | 17.91 | 3.39 | 2.11 | 3.16 |
| 81 | ARHGDIA Rho GDP-dissociation inhibitor 1 | IPI00003815 | 23 kDa | 0.047 | 9.04 | 21.39 | 13.78 | 4.24 | 3.17 | 5.53 |
| 82 | KRT15 Keratin, type I cytoskeletal 15 | IPI00290077 | 49 kDa | 0.81 | 95.39 | 77.67 | 151.57 | 41.55 | 76.14 | 172.33 |
| 83 | HBA1;HBA2 Hemoglobin subunit alpha | IPI00410714 | 15 kDa | 0.54 | 5.02 | 5.63 | 9.65 | 7.63 | 20.09 | 3.16 |
| 84 | PKP1 Isoform 2 of Plakophilin-1 | IPI00071509 | 83 kDa | 0.97 | 7.03 | 1 | 16.54 | 1 | 11.63 | 12.65 |
| 85 | ANXA6 annexin VI isoform 2 | IPI00002459 | 75 kDa | 0.14 | 4.02 | 18.01 | 15.16 | 1 | 7.4 | 3.16 |
| 86 | MFAP4 Microfibril-associated glycoprotein 4 | IPI00022792 | 29 kDa | 0.78 | 8.03 | 14.63 | 8.27 | 15.26 | 17.98 | 2.37 |
| 87 | KRT77 Keratin 77 | IPI00376379 | 62 kDa | 0.12 | 57.23 | 33.77 | 44.09 | 53.42 | 70.85 | 96.44 |
| 88 | ATP5A1 ATP synthase subunit alpha, mitochondrial | IPI00440493 | 60 kDa | 0.29 | 6.02 | 20.26 | 11.02 | 1 | 13.75 | 1 |
| 89 | GNB2L1 Lung cancer oncogene 7 | IPI00641950 | 38 kDa | 0.096 | 12.05 | 12.38 | 6.89 | 7.63 | 1.06 | 5.53 |
| 90 | OGN cDNA FLJ59205, highly similar to Mimecan | IPI00025465 | 41 kDa | 0.48 | 7.03 | 7.88 | 9.65 | 1.7 | 8.46 | 8.7 |
| 91 | YWHAE 14-3-3 protein epsilon | IPI00000816 | 29 kDa | 0.0051 | 20.08 | 28.14 | 27.56 | 8.48 | 1 | 8.7 |
| 92 | EEF2 Elongation factor 2 | IPI00186290 | 95 kDa | 0.68 | 11.05 | 3.38 | 8.27 | 1.7 | 9.52 | 7.11 |
| 93 | NME2 Nucleoside diphosphate kinase B | IPI00026260 | 17 kDa | 0.25 | 5.02 | 11.26 | 12.4 | 3.39 | 9.52 | 3.16 |
| 94 | CP Ceruloplasmin | IPI00017601 | 122 kDa | 0.58 | 5.02 | 2.25 | 6.89 | 22.89 | 3.17 | 1 |
| 95 | - 22 kDa protein | IPI00219910 | 22 kDa | 0.63 | 5.02 | 4.5 | 8.27 | 6.78 | 10.57 | 3.95 |
| 96 | YWHAB Isoform Long of 14-3-3 protein beta/alpha | IPI00216318 | 28 kDa | 0.0012 | 23.09 | 27.02 | 22.05 | 10.18 | 10.57 | 11.86 |
| 97 | - CDNA: FLJ23497 fis, clone LNG02604 | IPI00018048 | 16 kDa | 0.079 | 7.03 | 5.63 | 5.51 | 1 | 5.29 | 2.37 |
| 98 | APOA1 Apolipoprotein A-I | IPI00021841 | 31 kDa | 0.72 | 2.01 | 4.5 | 11.02 | 5.94 | 9.52 | 5.53 |
| 99 | ALDH1A1 Retinal dehydrogenase 1 | IPI00218914 | 55 kDa | 0.36 | 1 | 36.02 | 2.76 | 1 | 2.11 | 1 |
| 100 | PFN1 Profilin-1 | IPI00216691 | 15 kDa | 0.27 | 5.02 | 2.25 | 6.89 | 8.48 | 11.63 | 3.95 |
| 101 | GDI2 cDNA FLJ60299, highly similar to Rab GDP dissociation inhibitor beta | IPI00031461 | 51 kDa | 0.18 | 4.02 | 21.39 | 8.27 | 2.54 | 4.23 | 1 |
| 102 | ADH1B Alcohol dehydrogenase 1B | IPI00473031 | 40 kDa | 0.025 | 2.01 | 1 | 1.38 | 7.63 | 6.34 | 13.44 |
| 103 | GSTM3 Glutathione S-transferase Mu 3 | IPI00246975 | 27 kDa | 0.34 | 1 | 18.01 | 11.02 | 1 | 8.46 | 3.16 |
| 104 | DPT Dermatopontin | IPI00292130 | 24 kDa | 0.18 | 2.01 | 1 | 8.27 | 10.18 | 6.34 | 7.11 |
| 105 | FN1 Isoform 1 of Fibronectin | IPI00022418 | 263 kDa | 0.28 | 1 | 3.38 | 1.38 | 20.35 | 6.34 | 1 |
| 106 | HP;HPR haptoglobin isoform 2 preproprotein | IPI00478493 | 38 kDa | 0.25 | 4.02 | 2.25 | 1 | 16.11 | 7.4 | 1.58 |
| 107 | PHB Prohibitin | IPI00017334 | 30 kDa | 0.025 | 7.03 | 13.51 | 15.16 | 1 | 1 | 4.74 |
| 108 | GSTP1 Glutathione S-transferase P | IPI00219757 | 23 kDa | 0.51 | 5.02 | 9.01 | 5.51 | 6.78 | 6.34 | 2.37 |
| 109 | RPLP0 60S acidic ribosomal protein P0 | IPI00008530 | 34 kDa | 0.33 | 5.02 | 11.26 | 4.13 | 2.54 | 3.17 | 6.32 |
| 110 | MDH1 Malate dehydrogenase, cytoplasmic | IPI00291005 | 36 kDa | 0.62 | 5.02 | 12.38 | 2.76 | 3.39 | 6.34 | 5.53 |
| 111 | IGHM IGHM protein | IPI00549291 | 66 kDa | 0.079 | 2.01 | 1.13 | 2.76 | 5.09 | 13.75 | 6.32 |
| 112 | KRT82 Keratin type II cuticular Hb2 | IPI00300053 | 57 kDa | 0.16 | 6.02 | 1.13 | 4.13 | 4.24 | 11.63 | 8.7 |
| 113 | GNB1 Guanine nucleotide-binding protein G(I)/G(S)/G(T) subunit beta-1 | IPI00026268 | 37 kDa | 0.18 | 8.03 | 13.51 | 4.13 | 1 | 5.29 | 4.74 |
| 114 | PRDX6 Peroxiredoxin-6 | IPI00220301 | 25 kDa | 0.42 | 1 | 13.51 | 9.65 | 1 | 8.46 | 3.16 |
| 115 | CFL1 Cofilin-1 | IPI00012011 | 19 kDa | 0.83 | 6.02 | 5.63 | 5.51 | 5.09 | 7.4 | 3.95 |
| 116 | CAPZB cDNA, FLJ93598, highly similar to Homo sapiens capping protein (actin filament) muscle Z-line, beta (CAPZB), mRNA | IPI00641107 | 34 kDa | 0.017 | 8.03 | 6.75 | 9.65 | 2.54 | 1.06 | 4.74 |
| 117 | TALDO1 Transaldolase | IPI00744692 | 38 kDa | 0.14 | 3.01 | 16.88 | 9.65 | 3.39 | 1.06 | 2.37 |
| 118 | RNH1 Ribonuclease inhibitor | IPI00550069 | 50 kDa | 0.18 | 5.02 | 5.63 | 15.16 | 1 | 4.23 | 3.95 |
| 119 | MSN Moesin | IPI00219365 | 68 kDa | 0.25 | 7.03 | 3.38 | 12.4 | 1 | 6.34 | 3.16 |
| 120 | CTSD Cathepsin D | IPI00011229 | 45 kDa | 0.024 | 7.03 | 14.63 | 8.27 | 1 | 1.06 | 2.37 |
| 121 | HIST1H4F;HIST1H4J;HIST1H4A;HIST1H4C;HIST1H4E;HIST1H4L;HIST1H4K;HIST1H4I;HIST2H4B;HIST4H4;HIST1H4D;HIST1H4B;HIST1H4H;HIST2H4A Histone H4 | IPI00453473 | 11 kDa | 0.18 | 3.01 | 11.26 | 11.02 | 2.54 | 6.34 | 1 |
| 122 | ANXA4 annexin IV | IPI00793199 | 36 kDa | 0.13 | 6.02 | 7.88 | 6.89 | 4.24 | 1 | 6.32 |
| 123 | RAN GTP-binding nuclear protein Ran | IPI00643041 | 24 kDa | 0.21 | 5.02 | 6.75 | 11.02 | 1 | 6.34 | 4.74 |
| 124 | UBA1 Ubiquitin-like modifier-activating enzyme 1 | IPI00645078 | 118 kDa | 0.91 | 9.04 | 1 | 4.13 | 2.54 | 10.57 | 2.37 |
| 125 | SOD3 Extracellular superoxide dismutase [Cu-Zn] | IPI00027827 | 26 kDa | 0.88 | 3.01 | 2.25 | 9.65 | 6.78 | 1 | 8.7 |
| 126 | PEBP1 Phosphatidylethanolamine-binding protein 1 | IPI00219446 | 21 kDa | 0.026 | 10.04 | 6.75 | 8.27 | 1 | 5.29 | 2.37 |
| 127 | COL1A2 Collagen alpha-2(I) chain | IPI00304962 | 129 kDa | 1.00 | 10.04 | 4.5 | 1 | 6.78 | 6.34 | 2.37 |
| 128 | HRNR Hornerin | IPI00398625 | 282 kDa | 0.035 | 1 | 2.25 | 1 | 4.24 | 11.63 | 9.49 |
| 129 | - Similar to Keratin, type I cytoskeletal 10 | IPI00884222 | 57 kDa | 0.59 | 473.94 | 1 | 1 | 254.38 | 1 | 1 |
| 130 | DSG1 Desmoglein-1 | IPI00025753 | 114 kDa | 0.12 | 8.03 | 3.38 | 13.78 | 2.54 | 3.17 | 1 |
| 131 | PCBP1 Poly(rC)-binding protein 1 | IPI00016610 | 37 kDa | 0.075 | 6.02 | 11.26 | 5.51 | 1.7 | 3.17 | 3.95 |
| 132 | HNRNPA1 Isoform A1-B of Heterogeneous nuclear ribonucleoprotein A1 | IPI00215965 | 39 kDa | 0.091 | 9.04 | 9.01 | 2.76 | 1 | 2.11 | 3.16 |
| 133 | FLNA Isoform 2 of Filamin-A | IPI00302592 | 280 kDa | 0.077 | 15.06 | 7.88 | 4.13 | 1 | 2.11 | 1 |
| 134 | PSMA6 Proteasome subunit alpha type-6 | IPI00029623 | 27 kDa | 0.93 | 4.02 | 6.75 | 2.76 | 1.7 | 5.29 | 7.11 |
| 135 | RAB7A Ras-related protein Rab-7a | IPI00016342 | 23 kDa | 0.26 | 2.01 | 10.13 | 12.4 | 1 | 7.4 | 1 |
| 136 | VDAC1 Voltage-dependent anion-selective channel protein 1 | IPI00216308 | 31 kDa | 0.46 | 6.02 | 6.75 | 1.38 | 4.24 | 3.17 | 2.37 |
| 137 | DSC3 Isoform 3A of Desmocollin-3 | IPI00031549 | 100 kDa | 0.61 | 11.05 | 1 | 5.51 | 4.24 | 6.34 | 1.58 |
| 138 | YWHAQ 14-3-3 protein theta | IPI00018146 | 28 kDa | 0.00088 | 22.09 | 20.26 | 24.8 | 1 | 10.57 | 8.7 |
| 139 | FBLN1 Isoform D of Fibulin-1 | IPI00296534 | 77 kDa | 0.13 | 1 | 1.13 | 1 | 7.63 | 1.06 | 10.28 |
| 140 | IGHG4 IGHG4 protein | IPI00550640 | 52 kDa | 0.77 | 37.15 | 1 | 17.91 | 54.27 | 1 | 1 |
| 141 | CAPG Macrophage-capping protein | IPI00027341 | 39 kDa | 0.20 | 10.04 | 2.25 | 6.89 | 1.7 | 4.23 | 2.37 |
| 142 | NCOR2 nuclear receptor co-repressor 2 isoform 1 | IPI00001735 | 275 kDa | 0.33 | 1 | 2.25 | 5.51 | 1 | 2.11 | 1 |
| 143 | TUBB2C Tubulin beta-2C chain | IPI00007752 | 50 kDa | 0.075 | 48.2 | 106.94 | 1 | 6.78 | 15.86 | 13.44 |
| 144 | CAP1 Adenylyl cyclase-associated protein 1 | IPI00008274 | 52 kDa | 0.28 | 21.09 | 2.25 | 2.76 | 1 | 1.06 | 1 |
| 145 | KRT33A Keratin, type I cuticular Ha3-I | IPI00297632 | 46 kDa | 0.34 | 60.25 | 3.38 | 4.13 | 1 | 1 | 1 |
| 146 | HPX Hemopexin | IPI00022488 | 52 kDa | 0.85 | 5.02 | 1.13 | 5.51 | 10.18 | 1 | 2.37 |
| 147 | CBR1 Carbonyl reductase [NADPH] 1 | IPI00295386 | 30 kDa | 0.11 | 5.02 | 5.63 | 15.16 | 1.7 | 1 | 2.37 |
| 148 | PDIA3 Protein disulfide-isomerase A3 | IPI00025252 | 57 kDa | 0.012 | 10.04 | 6.75 | 5.51 | 1 | 2.11 | 1 |
| 149 | CLEC3B Tetranectin | IPI00009028 | 23 kDa | 0.35 | 1 | 2.25 | 5.51 | 1 | 10.57 | 7.11 |
| 150 | KRT12 Keratin, type I cytoskeletal 12 | IPI00015309 | 54 kDa | 0.60 | 36.15 | 30.39 | 1 | 26.29 | 30.67 | 128.85 |
| 151 | GC vitamin D-binding protein precursor | IPI00742696 | 53 kDa | 0.85 | 11.05 | 1 | 2.76 | 10.18 | 1.06 | 1 |
| 152 | VDAC2 Voltage-dependent anion-selective channel protein 2 | IPI00024145 | 38 kDa | 0.094 | 7.03 | 11.26 | 2.76 | 1.7 | 2.11 | 1 |
| 153 | VARS Valyl-tRNA synthetase | IPI00000873 | 140 kDa | 0.31 | 1 | 3.38 | 11.02 | 1 | 2.11 | 1.58 |
| 154 | F13A1 Coagulation factor XIII A chain | IPI00297550 | 83 kDa | 0.70 | 4.02 | 5.63 | 5.51 | 1 | 9.52 | 1 |
| 155 | ACTBL2 Beta-actin-like protein 2 | IPI00003269 | 42 kDa | 0.100 | 38.16 | 73.17 | 41.34 | 1 | 28.55 | 17.39 |
| 156 | APOB Apolipoprotein B-100 | IPI00022229 | 516 kDa | 0.12 | 1 | 1 | 1.38 | 9.33 | 9.52 | 1 |
| 157 | LGALS1 Galectin-1 | IPI00219219 | 15 kDa | 0.33 | 4.02 | 6.75 | 4.13 | 3.39 | 5.29 | 1.58 |
| 158 | VCP Transitional endoplasmic reticulum ATPase | IPI00022774 | 89 kDa | 0.82 | 2.01 | 10.13 | 1.38 | 2.54 | 6.34 | 2.37 |
| 159 | MYH4 Myosin-4 | IPI00001753 | 223 kDa | 0.31 | 3.01 | 3.38 | 1 | 1 | 1 | 2.37 |
| 160 | FLG Filaggrin | IPI00026256 | 435 kDa | 0.37 | 1 | 1 | 1 | 1 | 1 | 18.18 |
| 161 | S100A10 Protein S100-A10 | IPI00183695 | 11 kDa | 0.38 | 3.01 | 6.75 | 5.51 | 1.7 | 6.34 | 1.58 |
| 162 | RAC1 Isoform A of Ras-related C3 botulinum toxin substrate 1 | IPI00010271 | 21 kDa | 0.0092 | 5.02 | 6.75 | 8.27 | 1 | 2.11 | 2.37 |
| 163 | SPTAN1 Isoform 3 of Spectrin alpha chain, brain | IPI00843765 | 282 kDa | 0.25 | 1 | 6.75 | 12.4 | 1 | 4.23 | 1 |
| 164 | CFH Isoform 1 of Complement factor H | IPI00029739 | 139 kDa | 0.37 | 2.01 | 1.13 | 1.38 | 11.87 | 2.11 | 1 |
| 165 | - Ig kappa chain V-III region HIC | IPI00384576 | 14 kDa | 0.80 | 5.02 | 4.5 | 8.27 | 11.87 | 4.23 | 3.95 |
| 166 | HSPB1 Heat shock protein beta-1 | IPI00025512 | 23 kDa | 0.0027 | 6.02 | 6.75 | 8.27 | 1 | 2.11 | 2.37 |
| 167 | FASN Fatty acid synthase | IPI00026781 | 273 kDa | 0.023 | 7.03 | 6.75 | 4.13 | 1 | 3.17 | 1.58 |
| 168 | SERPINB5 Serpin B5 | IPI00783625 | 42 kDa | 0.51 | 5.02 | 1 | 9.65 | 1 | 3.17 | 5.53 |
| 169 | CSPG4 Chondroitin sulfate proteoglycan 4 | IPI00019157 | 250 kDa | 0.026 | 2.01 | 3.38 | 2.76 | 1 | 1 | 1.58 |
| 170 | HSP90AB1 Heat shock protein HSP 90-beta | IPI00414676 | 83 kDa | 0.013 | 17.07 | 27.02 | 17.91 | 4.24 | 8.46 | 4.74 |
| 171 | PSMB6 Proteasome subunit beta type-6 | IPI00000811 | 25 kDa | 0.75 | 2.01 | 4.5 | 5.51 | 1.7 | 2.11 | 6.32 |
| 172 | C4A Complement C4-A | IPI00032258 | 193 kDa | 0.34 | 1 | 1 | 1 | 13.57 | 1.06 | 1.58 |
| 173 | PRDX4 Peroxiredoxin-4 | IPI00011937 | 31 kDa | 0.81 | 1 | 6.75 | 2.76 | 7.63 | 3.17 | 1.58 |
| 174 | VCL Isoform 1 of Vinculin | IPI00291175 | 117 kDa | 0.32 | 9.04 | 1 | 4.13 | 1.7 | 3.17 | 1 |
| 175 | CASP14 Caspase-14 | IPI00013885 | 28 kDa | 0.62 | 1 | 1 | 6.89 | 4.24 | 3.17 | 4.74 |
| 176 | TTLL3;ARPC4 Actin-related protein 2/3 complex subunit 4 | IPI00554811 | 20 kDa | 0.51 | 3.01 | 1.13 | 4.13 | 2.54 | 7.4 | 2.37 |
| 177 | APRT Adenine phosphoribosyltransferase | IPI00218693 | 20 kDa | 0.87 | 2.01 | 2.25 | 6.89 | 1.7 | 5.29 | 3.16 |
| 178 | DNAH1 Isoform 2 of Dynein heavy chain 1, axonemal | IPI00002127 | 486 kDa | 0.17 | 1 | 3.38 | 4.13 | 1.7 | 1 | 1 |
| 179 | HNRNPA2B1 Isoform B1 of Heterogeneous nuclear ribonucleoproteins A2/B1 | IPI00396378 | 37 kDa | 0.015 | 10.04 | 10.13 | 5.51 | 1 | 1.06 | 3.16 |
| 180 | MKI67 Isoform Long of Antigen KI-67 | IPI00004233 | 359 kDa | 0.34 | 3.01 | 1 | 1.38 | 3.39 | 8.46 | 1 |
| 181 | IGL@ IGL@ protein | IPI00719373 | 23 kDa | 0.22 | 10.04 | 10.13 | 12.4 | 27.13 | 20.09 | 8.7 |
| 182 | ##IPI00221255 | ##IPI00221255 | ? | 0.0018 | 3.01 | 2.25 | 2.76 | 1 | 1.06 | 1 |
| 183 | FLG2 Filaggrin-2 | IPI00397801 | 248 kDa | 0.000021 | 1 | 1 | 1.38 | 5.09 | 5.29 | 5.53 |
| 184 | EIF4A1 Eukaryotic initiation factor 4A-I | IPI00025491 | 46 kDa | 0.21 | 3.01 | 9.01 | 2.76 | 1 | 2.11 | 2.37 |
| 185 | GPNMB Isoform 2 of Transmembrane glycoprotein NMB | IPI00001592 | 63 kDa | 0.079 | 11.05 | 3.38 | 5.51 | 1 | 1.06 | 1 |
| 186 | TKT cDNA FLJ54957, highly similar to Transketolase | IPI00643920 | 69 kDa | 0.46 | 1 | 13.51 | 1 | 1 | 3.17 | 1 |
| 187 | IGHA1;IGHV3OR16-13 IGHA1 protein | IPI00430842 | 53 kDa | 0.57 | 5.02 | 1 | 1 | 12.72 | 1 | 1 |
| 188 | DSTN Destrin | IPI00473014 | 19 kDa | 0.27 | 7.03 | 2.25 | 4.13 | 1.7 | 4.23 | 1 |
| 189 | YWHAG 14-3-3 protein gamma | IPI00220642 | 28 kDa | 0.024 | 15.06 | 21.39 | 24.8 | 9.33 | 11.63 | 7.91 |
| 190 | RYR3 Uncharacterized protein RYR3 | IPI00217185 | 552 kDa | 0.013 | 2.01 | 3.38 | 2.76 | 1 | 1.06 | 1 |
| 191 | GPI Glucose-6-phosphate isomerase | IPI00027497 | 63 kDa | 0.46 | 5.02 | 4.5 | 1 | 1.7 | 3.17 | 2.37 |
| 192 | SEPT2 Septin-2 | IPI00014177 | 41 kDa | 0.28 | 3.01 | 9.01 | 1.38 | 2.54 | 1 | 1 |
| 193 | - Ig kappa chain V-IV region B17 | IPI00386133 | 15 kDa | 0.57 | 2.01 | 2.25 | 2.76 | 9.33 | 1 | 1.58 |
| 194 | TMED10 Transmembrane emp24 domain-containing protein 10 | IPI00028055 | 25 kDa | 0.15 | 1 | 1.13 | 2.76 | 5.94 | 5.29 | 1.58 |
| 195 | TLN1 Talin-1 | IPI00298994 | 270 kDa | 0.21 | 4.02 | 10.13 | 1 | 1 | 1.06 | 1 |
| 196 | GPX3 Glutathione peroxidase 3 | IPI00026199 | 26 kDa | 0.083 | 2.01 | 5.63 | 6.89 | 1 | 2.11 | 1 |
| 197 | FABP5L7;FABP5 Fatty acid-binding protein, epidermal | IPI00007797 | 15 kDa | 0.58 | 9.04 | 1 | 1.38 | 2.54 | 1 | 3.16 |
| 198 | DCD Dermcidin | IPI00027547 | 11 kDa | 0.53 | 1 | 1 | 5.51 | 1 | 4.23 | 7.11 |
| 199 | HIST2H2BE Histone H2B type 2-E | IPI00003935 | 14 kDa | 0.99 | 2.01 | 2.25 | 4.13 | 2.54 | 4.23 | 1.58 |
| 200 | PSMB1 Proteasome subunit beta type-1 | IPI00025019 | 26 kDa | 0.045 | 3.01 | 4.5 | 6.89 | 1 | 2.11 | 1 |
| 201 | EPPK1 Epiplakin | IPI00010951 | 556 kDa | 0.63 | 2.01 | 1 | 4.13 | 1.7 | 4.23 | 3.16 |
| 202 | PSMB5 Proteasome subunit beta type-5 | IPI00479306 | 28 kDa | 0.95 | 1 | 4.5 | 2.76 | 1.7 | 2.11 | 4.74 |
| 203 | PSMA4 Proteasome subunit alpha type-4 | IPI00299155 | 29 kDa | 0.20 | 3.01 | 5.63 | 2.76 | 3.39 | 1 | 1.58 |
| 204 | RAB1B Ras-related protein Rab-1B | IPI00008964 | 22 kDa | 0.77 | 10.04 | 20.26 | 1 | 20.35 | 22.21 | 3.16 |
| 205 | ESD S-formylglutathione hydrolase | IPI00411706 | 31 kDa | 0.14 | 1 | 6.75 | 8.27 | 1.7 | 1 | 1 |
| 206 | ALDH2 Aldehyde dehydrogenase, mitochondrial | IPI00006663 | 56 kDa | 0.34 | 7.03 | 4.5 | 1 | 1 | 3.17 | 2.37 |
| 207 | GRIK2 Isoform 1 of Glutamate receptor, ionotropic kainate 2 | IPI00011396 | 103 kDa | 0.061 | 2.01 | 3.38 | 5.51 | 1 | 1 | 1 |
| 208 | PHGDH D-3-phosphoglycerate dehydrogenase | IPI00011200 | 57 kDa | 0.031 | 7.03 | 5.63 | 2.76 | 1 | 1.06 | 1 |
| 209 | CFB cDNA FLJ55673, highly similar to Complement factor B | IPI00019591 | 141 kDa | 0.34 | 1 | 1 | 1 | 11.02 | 1 | 1.58 |
| 210 | RAB2A Ras-related protein Rab-2A | IPI00031169 | 24 kDa | 0.092 | 2.01 | 4.5 | 5.51 | 1.7 | 2.11 | 1 |
| 211 | AHNAK Neuroblast differentiation-associated protein AHNAK | IPI00021812 | 629 kDa | 0.16 | 3.01 | 5.63 | 1.38 | 1 | 1.06 | 1.58 |
| 212 | PRDX3 Thioredoxin-dependent peroxide reductase, mitochondrial | IPI00024919 | 28 kDa | 0.36 | 3.01 | 2.25 | 5.51 | 1 | 4.23 | 1 |
| 213 | ATP5F1 ATP synthase subunit b, mitochondrial | IPI00029133 | 29 kDa | 0.87 | 2.01 | 1 | 5.51 | 1 | 5.29 | 3.16 |
| 214 | AKR1B1 Aldose reductase | IPI00413641 | 36 kDa | 0.25 | 3.01 | 12.38 | 1 | 1 | 1 | 1 |
| 215 | ACTN1 Alpha-actinin-1 | IPI00013508 | 103 kDa | 0.43 | 22.09 | 1 | 11.02 | 1 | 1 | 5.53 |
| 216 | - cDNA FLJ61158, highly similar to ADP-ribosylation factor-like protein 8B | IPI00789069 | 21 kDa | 0.47 | 1 | 3.38 | 1.38 | 1.7 | 4.23 | 2.37 |
| 217 | PSMA7 Isoform 1 of Proteasome subunit alpha type-7 | IPI00024175 | 28 kDa | 0.061 | 4.02 | 2.25 | 5.51 | 1.7 | 1 | 1.58 |
| 218 | HSD17B10 Isoform 1 of 3-hydroxyacyl-CoA dehydrogenase type-2 | IPI00017726 | 27 kDa | 0.22 | 3.01 | 7.88 | 1.38 | 1.7 | 1 | 1 |
| 219 | CLIC1 Chloride intracellular channel protein 1 | IPI00010896 | 27 kDa | 0.015 | 3.01 | 4.5 | 5.51 | 1.7 | 1 | 1 |
| 220 | COL7A1 Isoform 2 of Collagen alpha-1(VII) chain | IPI00795118 | 292 kDa | 0.91 | 3.01 | 2.25 | 1.38 | 1 | 4.23 | 1 |
| 221 | COX4I1 Cytochrome c oxidase subunit 4 isoform 1, mitochondrial | IPI00006579 | 20 kDa | 0.65 | 2.01 | 1 | 2.76 | 1 | 5.29 | 1.58 |
| 222 | KNTC1 Kinetochore-associated protein 1 | IPI00001458 | 251 kDa | 0.96 | 1 | 2.25 | 1 | 2.54 | 1 | 1 |
| 223 | ATP5O ATP synthase subunit O, mitochondrial | IPI00007611 | 23 kDa | 0.56 | 1 | 4.5 | 4.13 | 1 | 4.23 | 1.58 |
| 224 | SOD1 Superoxide dismutase [Cu-Zn] | IPI00218733 | 16 kDa | 0.54 | 2.01 | 1 | 8.27 | 2.54 | 3.17 | 1 |
| 225 | LOX Protein-lysine 6-oxidase | IPI00002802 | 47 kDa | 0.33 | 1 | 1 | 1.38 | 5.09 | 1 | 1.58 |
| 226 | PRELP Prolargin | IPI00020987 | 44 kDa | 0.39 | 1 | 1 | 1 | 1 | 1 | 11.07 |
| 227 | CAPN1 Calpain-1 catalytic subunit | IPI00011285 | 82 kDa | 0.53 | 1 | 1.13 | 2.76 | 1 | 5.29 | 1.58 |
| 228 | HSPA5 HSPA5 protein | IPI00003362 | 72 kDa | 0.25 | 3.01 | 2.25 | 6.89 | 1 | 3.17 | 1.58 |
| 229 | PSMB4 Proteasome subunit beta type-4 | IPI00555956 | 29 kDa | 0.38 | 1 | 3.38 | 4.13 | 1 | 2.11 | 2.37 |
| 230 | CDC42 Isoform 2 of Cell division control protein 42 homolog | IPI00016786 | 21 kDa | 0.017 | 6.02 | 4.5 | 6.89 | 1 | 2.11 | 3.16 |
| 231 | CAPZA2 F-actin-capping protein subunit alpha-2 | IPI00026182 | 33 kDa | 0.77 | 2.01 | 3.38 | 1 | 1.7 | 2.11 | 2.37 |
| 232 | GANAB cDNA FLJ61290, highly similar to Neutral alpha-glucosidase AB | IPI00383581 | 113 kDa | 0.91 | 5.02 | 1 | 1.38 | 4.24 | 1 | 1.58 |
| 233 | CA2 Carbonic anhydrase 2 | IPI00218414 | 29 kDa | 0.53 | 3.01 | 1 | 4.13 | 1.7 | 3.17 | 1 |
| 234 | ACTR3 Actin-related protein 3 | IPI00028091 | 47 kDa | 0.11 | 7.03 | 4.5 | 1.38 | 1 | 1.06 | 1 |
| 235 | TUBA1B Tubulin alpha-1B chain | IPI00387144 | 50 kDa | 0.25 | 24.1 | 70.92 | 1 | 11.02 | 16.92 | 1 |
| 236 | FLNB Isoform 1 of Filamin-B | IPI00289334 | 278 kDa | 0.27 | 8.03 | 2.25 | 1 | 1 | 1 | 1 |
| 237 | UQCRC2 Cytochrome b-c1 complex subunit 2, mitochondrial | IPI00305383 | 48 kDa | 0.35 | 1 | 12.38 | 1.38 | 1 | 1 | 1 |
| 238 | DSC1 Isoform 1A of Desmocollin-1 | IPI00216099 | 100 kDa | 0.52 | 1 | 1 | 2.76 | 1 | 1 | 7.91 |
| 239 | GCN1L1 Translational activator GCN1 | IPI00001159 | 293 kDa | 0.36 | 1 | 1.13 | 5.51 | 1 | 1 | 1 |
| 240 | ARF5 ADP-ribosylation factor 5 | IPI00215919 | 21 kDa | 0.72 | 1 | 1.13 | 5.51 | 1 | 3.17 | 1.58 |
| 241 | HTT Huntingtin | IPI00002335 | 348 kDa | 0.21 | 1 | 3.38 | 8.27 | 1 | 1.06 | 1 |
| 242 | HNRNPH1 51 kDa protein | IPI00479191 | 51 kDa | 0.20 | 3.01 | 6.75 | 1 | 1 | 1.06 | 1 |
| 243 | ARG1 Isoform 3 of Arginase-1 | IPI00038356 | 25 kDa | 0.70 | 2.01 | 2.25 | 1.38 | 1 | 1 | 5.53 |
| 244 | SPTBN1 Isoform Long of Spectrin beta chain, brain 1 | IPI00005614 | 275 kDa | 0.22 | 5.02 | 3.38 | 1 | 1 | 2.11 | 1 |
| 245 | ACAT1 Acetyl-CoA acetyltransferase, mitochondrial | IPI00030363 | 45 kDa | 0.23 | 1 | 3.38 | 8.27 | 1 | 1 | 1.58 |
| 246 | HSPD1 60 kDa heat shock protein, mitochondrial | IPI00784154 | 61 kDa | 0.41 | 1 | 9.01 | 1 | 1 | 2.11 | 1 |
| 247 | CMA1 Chymase | IPI00013937 | 27 kDa | 0.91 | 4.02 | 1 | 1 | 4.24 | 1 | 1.58 |
| 248 | CRYL1 Isoform 1 of Lambda-crystallin homolog | IPI00006443 | 35 kDa | 0.26 | 1 | 10.13 | 2.76 | 1 | 1 | 1 |
| 249 | IGKV1-5 IGKV1-5 protein | IPI00419424 | 26 kDa | 0.62 | 21.09 | 39.4 | 46.85 | 94.12 | 34.9 | 17.39 |
| 250 | IDH1 Isocitrate dehydrogenase [NADP] cytoplasmic | IPI00027223 | 47 kDa | 0.19 | 1 | 3.38 | 5.51 | 1 | 1.06 | 1.58 |
| 251 | - Uncharacterized protein ENSP00000374805 | IPI00854644 | 13 kDa | 0.91 | 2.01 | 2.25 | 2.76 | 4.24 | 2.11 | 1 |
| 252 | PARK7 Protein DJ-1 | IPI00298547 | 20 kDa | 0.18 | 1 | 4.5 | 5.51 | 1 | 2.11 | 1 |
| 253 | RPSA 33 kDa protein | IPI00413108 | 33 kDa | 0.024 | 4.02 | 2.25 | 4.13 | 1 | 1 | 1.58 |
| 254 | HSP90B1 Endoplasmin | IPI00027230 | 92 kDa | 0.13 | 8.03 | 1 | 6.89 | 1.7 | 1.06 | 1 |
| 255 | CTSB Cathepsin B | IPI00295741 | 38 kDa | 0.35 | 3.01 | 3.38 | 1 | 1.7 | 2.11 | 1 |
| 256 | AHCY Adenosylhomocysteinase | IPI00012007 | 48 kDa | 0.91 | 1 | 4.5 | 1 | 5.09 | 1.06 | 1 |
| 257 | HBG1 Hemoglobin subunit gamma-1 | IPI00220706 | 16 kDa | 0.30 | 1 | 1 | 1 | 5.09 | 16.92 | 1 |
| 258 | APOH Beta-2-glycoprotein 1 | IPI00298828 | 38 kDa | 0.44 | 8.03 | 1 | 1 | 2.54 | 1 | 1 |
| 259 | KRT38 Keratin, type I cuticular Ha8 | IPI00297641 | 50 kDa | 0.28 | 36.15 | 1 | 1 | 1 | 1 | 1 |
| 260 | ACTR2 Actin-related protein 2 | IPI00005159 | 45 kDa | 0.55 | 1 | 2.25 | 2.76 | 1.7 | 2.11 | 1 |
| 261 | KRT6B Keratin, type II cytoskeletal 6B | IPI00293665 | 60 kDa | 0.90 | 213.87 | 122.7 | 118.5 | 1 | 143.82 | 182.61 |
| 262 | DPYSL2 Dihydropyrimidinase-related protein 2 | IPI00257508 | 62 kDa | 0.25 | 2.01 | 5.63 | 1.38 | 1 | 1 | 1.58 |
| 263 | GNAI3 Guanine nucleotide-binding protein G(k) subunit alpha | IPI00220578 | 41 kDa | 0.28 | 3.01 | 1 | 1.38 | 1 | 1.06 | 1 |
| 264 | C1QC Complement C1q subcomponent subunit C | IPI00022394 | 26 kDa | 0.52 | 1 | 1.13 | 2.76 | 3.39 | 1 | 2.37 |
| 265 | MYL6B Myosin light chain 6B | IPI00027255 | 23 kDa | 0.19 | 1 | 4.5 | 4.13 | 1 | 2.11 | 1 |
| 266 | ARPC2 Actin-related protein 2/3 complex subunit 2 | IPI00005161 | 34 kDa | 0.14 | 4.02 | 1.13 | 5.51 | 1 | 1 | 1.58 |
| 267 | ##IPI00651691 | ##IPI00651691 | ? | 0.0040 | 2.01 | 2.25 | 2.76 | 1 | 1.06 | 1 |
| 268 | PKM2 cDNA FLJ53368, highly similar to Pyruvate kinase isozymes M1/M2 | IPI00847989 | 50 kDa | 0.64 | 31.13 | 7.88 | 1 | 1 | 9.52 | 15.81 |
| 269 | CRYZ Quinone oxidoreductase | IPI00000792 | 35 kDa | 0.31 | 2.01 | 6.75 | 1 | 1 | 1 | 1.58 |
| 270 | UQCRC1 Cytochrome b-c1 complex subunit 1, mitochondrial | IPI00013847 | 53 kDa | 0.23 | 1 | 3.38 | 5.51 | 1 | 2.11 | 1 |
| 271 | AOC3 Membrane copper amine oxidase | IPI00004457 | 85 kDa | 0.83 | 4.02 | 1 | 1 | 1 | 3.17 | 1 |
| 272 | LAMA4 Isoform 1 of Laminin subunit alpha-4 | IPI00329482 | 203 kDa | 0.79 | 1 | 4.5 | 1 | 1 | 6.34 | 1 |
| 273 | GSTO1 Glutathione transferase omega-1 | IPI00019755 | 28 kDa | 0.25 | 1 | 9.01 | 2.76 | 1 | 1 | 1 |
| 274 | TUFM Tu translation elongation factor, mitochondrial precursor | IPI00027107 | 50 kDa | 0.37 | 1 | 11.26 | 1 | 1 | 1 | 1 |
| 275 | - cDNA FLJ76502, highly similar to Homo sapiens ubiquinol-cytochrome c reductase, Rieske iron-sulfur polypeptide 1 (UQCRFS1), mRNA | IPI00883602 | 30 kDa | 0.64 | 1 | 2.25 | 1.38 | 1 | 2.11 | 2.37 |
| 276 | SOD2 Superoxide dismutase [Mn], mitochondrial | IPI00022314 | 25 kDa | 0.16 | 1 | 3.38 | 5.51 | 1 | 1.06 | 1 |
| 277 | CPA3 Mast cell carboxypeptidase A | IPI00009829 | 49 kDa | 0.16 | 5.02 | 2.25 | 1.38 | 1 | 1 | 1 |
| 278 | ECH1 Delta(3,5)-Delta(2,4)-dienoyl-CoA isomerase, mitochondrial | IPI00011416 | 36 kDa | 0.00034 | 3.01 | 3.38 | 2.76 | 1 | 1 | 1 |
| 279 | TGFBI Transforming growth factor-beta-induced protein ig-h3 | IPI00018219 | 75 kDa | 0.67 | 1 | 4.5 | 1 | 1.7 | 2.11 | 1 |
| 280 | RAB5B Ras-related protein Rab-5B | IPI00017344 | 24 kDa | 0.45 | 1 | 5.63 | 1 | 1.7 | 1.06 | 1 |
| 281 | ETFA Electron transfer flavoprotein subunit alpha, mitochondrial | IPI00010810 | 35 kDa | 0.0028 | 4.02 | 3.38 | 2.76 | 1 | 1 | 1 |
| 282 | CCT7 T-complex protein 1 subunit eta | IPI00018465 | 59 kDa | 0.42 | 5.02 | 1.13 | 1 | 1 | 1 | 1.58 |
| 283 | CCT2 T-complex protein 1 subunit beta | IPI00297779 | 57 kDa | 0.13 | 3.01 | 5.63 | 1.38 | 1 | 1 | 1 |
| 284 | FBLN5 Fibulin-5 | IPI00294615 | 50 kDa | 0.0028 | 4.02 | 3.38 | 2.76 | 1 | 1 | 1 |
| 285 | KRTAP11-1 Keratin-associated protein 11-1 | IPI00216711 | 17 kDa | 0.21 | 7.03 | 1 | 2.76 | 1 | 1 | 1 |
| 286 | KRT25 Keratin, type I cytoskeletal 25 | IPI00375911 | 49 kDa | 0.78 | 30.12 | 21.39 | 1 | 1 | 1 | 1 |
| 287 | ARHGDIB Rho GDP-dissociation inhibitor 2 | IPI00003817 | 23 kDa | 0.93 | 1 | 2.25 | 1 | 1 | 1.06 | 2.37 |
| 288 | SAR1A GTP-binding protein SAR1a | IPI00015954 | 22 kDa | 0.89 | 1 | 2.25 | 1.38 | 1 | 1 | 2.37 |
| 289 | CAND1 Isoform 1 of Cullin-associated NEDD8-dissociated protein 1 | IPI00100160 | 136 kDa | 0.24 | 3.01 | 1 | 1.38 | 1 | 1.06 | 1 |
| 290 | HNRNPU Isoform Short of Heterogeneous nuclear ribonucleoprotein U | IPI00479217 | 89 kDa | 0.0037 | 2.01 | 2.25 | 2.76 | 1 | 1 | 1 |
| 291 | MYH3 Myosin-3 | IPI00298301 | 224 kDa | 0.40 | 1 | 1.13 | 1 | 1 | 3.17 | 1 |
| 292 | ABHD14B Isoform 1 of Abhydrolase domain-containing protein 14B | IPI00063827 | 22 kDa | 0.85 | 2.01 | 2.25 | 1.38 | 1 | 3.17 | 1 |
| 293 | PPP2R1A cDNA FLJ34068 fis, clone FCBBF3001918, highly similar to SERINE/THREONINE PROTEIN PHOSPHATASE 2A, 65 kDa REGULATORY SUBUNIT A, ALPHA ISOFORM | IPI00168184 | 57 kDa | 0.73 | 1 | 2.25 | 1 | 1 | 3.17 | 1 |
| 294 | RAB11B Ras-related protein Rab-11B | IPI00020436 | 24 kDa | 0.54 | 1 | 3.38 | 1.38 | 1 | 2.11 | 1 |
| 295 | KPNB1 Importin subunit beta-1 | IPI00001639 | 97 kDa | 0.36 | 4.02 | 1.13 | 1 | 1 | 1.06 | 1 |
| 296 | RPL11 Isoform 1 of 60S ribosomal protein L11 | IPI00376798 | 20 kDa | 0.20 | 1 | 4.5 | 2.76 | 1 | 1 | 1.58 |
| 297 | SEPT7 Isoform 1 of Septin-7 | IPI00033025 | 51 kDa | 0.43 | 1 | 5.63 | 1 | 1 | 1 | 1.58 |
| 298 | DECR1 2,4-dienoyl-CoA reductase, mitochondrial | IPI00003482 | 36 kDa | 0.33 | 1 | 6.75 | 1.38 | 1 | 1 | 1 |
| 299 | EIF6 Eukaryotic translation initiation factor 6 | IPI00010105 | 27 kDa | 0.15 | 1 | 3.38 | 5.51 | 1 | 1 | 1 |
| 300 | KCTD12 BTB/POZ domain-containing protein KCTD12 | IPI00060715 | 36 kDa | 0.42 | 1 | 3.38 | 1 | 1.7 | 1 | 1 |
| 301 | EIF3A Eukaryotic translation initiation factor 3 subunit A | IPI00029012 | 167 kDa | 0.38 | 1 | 5.63 | 1 | 1 | 1.06 | 1 |
| 302 | IGKC IGKC protein | IPI00845354 | 25 kDa | 0.18 | 1 | 1 | 1 | 6.78 | 4.23 | 1 |
| 303 | SPTB Isoform 2 of Spectrin beta chain, erythrocyte | IPI00216704 | 268 kDa | 0.37 | 1 | 1 | 1 | 1 | 7.4 | 1 |
| 304 | TUBB4 Tubulin beta-4 chain | IPI00023598 | 50 kDa | 0.11 | 1 | 120.44 | 1 | 1 | 1 | 1 |
| 305 | SDCBP Syntenin-1 | IPI00299086 | 32 kDa | 0.34 | 1 | 7.88 | 1 | 1 | 1 | 1 |
| 306 | CAPZA1 F-actin-capping protein subunit alpha-1 | IPI00005969 | 33 kDa | 0.0044 | 4.02 | 3.38 | 4.13 | 1 | 1 | 2.37 |
| 307 | LAMB4 Isoform 1 of Laminin subunit beta-4 | IPI00295437 | 194 kDa | 0.42 | 2.01 | 1 | 1.38 | 1 | 1.06 | 1.58 |
| 308 | NDUFS3 NADH dehydrogenase [ubiquinone] iron-sulfur protein 3, mitochondrial | IPI00025796 | 30 kDa | 0.030 | 2.01 | 2.25 | 1 | 1 | 1.06 | 1 |
| 309 | P4HB Protein disulfide-isomerase | IPI00010796 | 57 kDa | 0.14 | 3.01 | 2.25 | 1 | 1 | 1.06 | 1 |
| 310 | BDH2 Isoform 1 of 3-hydroxybutyrate dehydrogenase type 2 | IPI00607799 | 27 kDa | 0.31 | 1 | 4.5 | 1.38 | 1 | 1.06 | 1 |
| 311 | PHB2 Prohibitin-2 | IPI00027252 | 33 kDa | 0.49 | 3.01 | 1.13 | 1 | 1 | 1 | 1.58 |
| 312 | GNAI2 Isoform 2 of Guanine nucleotide-binding protein G(i), alpha-2 subunit | IPI00217906 | 38 kDa | 0.56 | 1 | 2.25 | 4.13 | 1 | 3.17 | 1 |
| 313 | NPEPPS Puromycin-sensitive aminopeptidase | IPI00026216 | 103 kDa | 0.60 | 2.01 | 1 | 1 | 3.39 | 1.06 | 1 |
| 314 | PDIA4 Protein disulfide-isomerase A4 | IPI00009904 | 73 kDa | 0.44 | 1 | 1 | 5.51 | 1 | 1.06 | 1.58 |
| 315 | SERPINC1 Antithrombin III variant | IPI00032179 | 53 kDa | 0.44 | 1 | 1 | 1.38 | 3.39 | 1.06 | 1 |
| 316 | TYMP Thymidine phosphorylase | IPI00292858 | 50 kDa | 0.23 | 3.01 | 2.25 | 1 | 1 | 1 | 1.58 |
| 317 | APCS Serum amyloid P-component | IPI00022391 | 25 kDa | 0.38 | 1 | 1 | 6.89 | 1 | 1 | 1 |
| 318 | MAPK1 Mitogen-activated protein kinase 1 | IPI00003479 | 41 kDa | 0.36 | 1 | 1.13 | 5.51 | 1 | 1.06 | 1 |
| 319 | LAMC1 Laminin subunit gamma-1 | IPI00298281 | 178 kDa | 0.38 | 1 | 5.63 | 1 | 1 | 1.06 | 1 |
| 320 | HERC2 Probable E3 ubiquitin-protein ligase HERC2 | IPI00005826 | 527 kDa | 0.22 | 1 | 2.25 | 1.38 | 1 | 1 | 1 |
| 321 | GOT2 Aspartate aminotransferase, mitochondrial | IPI00018206 | 47 kDa | 0.12 | 1 | 4.5 | 4.13 | 1 | 1 | 1 |
| 322 | GAPDHS Glyceraldehyde-3-phosphate dehydrogenase, testis-specific | IPI00022430 | 45 kDa | 0.33 | 1 | 6.75 | 1.38 | 1 | 1 | 1 |
| 323 | CTHRC1 Isoform 1 of Collagen triple helix repeat-containing protein 1 | IPI00060423 | 26 kDa | 0.41 | 1 | 1 | 1 | 1 | 1 | 4.74 |
| 324 | SERPINF1 Pigment epithelium-derived factor | IPI00006114 | 46 kDa | 0.37 | 1 | 1 | 1 | 1 | 1 | 4.74 |
| 325 | IQGAP1 Ras GTPase-activating-like protein IQGAP1 | IPI00009342 | 189 kDa | 0.38 | 1 | 3.38 | 1 | 1 | 1.06 | 1 |
| 326 | LAMB2 Laminin subunit beta-2 | IPI00296922 | 196 kDa | 0.74 | 1 | 2.25 | 1 | 1.7 | 1.06 | 1 |
| 327 | TAGLN Transgelin | IPI00216138 | 23 kDa | 0.49 | 1 | 1.13 | 1.38 | 1 | 3.17 | 1 |
| 328 | CCT4 T-complex protein 1 subunit delta | IPI00302927 | 58 kDa | 0.29 | 1 | 3.38 | 1 | 1 | 1.06 | 1 |
| 329 | UBC;UBB;RPS27A ubiquitin and ribosomal protein S27a precursor | IPI00179330 | 18 kDa | 0.29 | 1 | 3.38 | 1 | 1 | 1.06 | 1 |
| 330 | ACO2 Aconitate hydratase, mitochondrial | IPI00017855 | 85 kDa | 0.38 | 1 | 2.25 | 2.76 | 1 | 2.11 | 1 |
| 331 | HLA-C;HLA-B;MICA;XXbac-BPG181B23.1 HLA class I histocompatibility antigen, B-50 alpha chain | IPI00471955 | 41 kDa | 0.26 | 2.01 | 1 | 2.76 | 1 | 1 | 1.58 |
| 332 | NEB Nebulin | IPI00303335 | 773 kDa | 0.21 | 1 | 2.25 | 1.38 | 1 | 1 | 1 |
| 333 | ADH5P4 similar to rcADH5 | IPI00176678 | 41 kDa | 0.062 | 3.01 | 2.25 | 1 | 1 | 1 | 1 |
| 334 | KRT73 Isoform 1 of Keratin, type II cytoskeletal 73 | IPI00174775 | 59 kDa | 0.59 | 1 | 30.39 | 1 | 16.11 | 1 | 126.48 |
| 335 | HNRNPC Isoform C1 of Heterogeneous nuclear ribonucleoproteins C1/C2 | IPI00216592 | 32 kDa | 0.13 | 1 | 3.38 | 2.76 | 1 | 1 | 1 |
| 336 | IMMT Isoform 1 of Mitochondrial inner membrane protein | IPI00009960 | 84 kDa | 0.37 | 1 | 1 | 1 | 1 | 3.17 | 1 |
| 337 | PHRF1 Isoform 1 of RING and PHD-finger domain-containing protein KIAA1542 | IPI00001813 | 179 kDa | 0.33 | 1 | 1.13 | 2.76 | 1 | 1 | 1 |
| 338 | RPS12 40S ribosomal protein S12 | IPI00013917 | 15 kDa | 0.89 | 1 | 1 | 2.76 | 1 | 3.17 | 1 |
| 339 | HNRNPR Heterogeneous nuclear ribonucleoprotein R | IPI00012074 | 71 kDa | 0.32 | 1 | 1 | 1 | 1 | 1.06 | 1.58 |
| 340 | RAB5C Ras-related protein Rab-5C | IPI00016339 | 23 kDa | 0.13 | 1 | 5.63 | 4.13 | 1 | 1 | 1 |
| 341 | ARPC3 Actin-related protein 2/3 complex subunit 3 | IPI00005162 | 21 kDa | 0.95 | 1 | 3.38 | 1 | 1 | 3.17 | 1 |
| 342 | RAB14 Ras-related protein Rab-14 | IPI00291928 | 24 kDa | 0.12 | 4.02 | 1 | 2.76 | 1 | 1 | 1 |
| 343 | AMBP AMBP protein | IPI00022426 | 39 kDa | 0.37 | 1 | 1 | 1 | 1 | 1 | 3.95 |
| 344 | HNRNPK Isoform 1 of Heterogeneous nuclear ribonucleoprotein K | IPI00216049 | 51 kDa | 0.38 | 1 | 5.63 | 1 | 1 | 1 | 1 |
| 345 | KRT81 Keratin type II cuticular Hb1 | IPI00760863 | 55 kDa | 0.33 | 183.75 | 1 | 1 | 1 | 1 | 1 |
| 346 | NAPA Alpha-soluble NSF attachment protein | IPI00009253 | 33 kDa | 0.30 | 2.01 | 1.13 | 1 | 1 | 1 | 1 |
| 347 | FH Isoform Mitochondrial of Fumarate hydratase, mitochondrial | IPI00296053 | 55 kDa | 0.85 | 1 | 1.13 | 1.38 | 1 | 1 | 1.58 |
| 348 | SERPINB6 Putative uncharacterized protein DKFZp686I04222 | IPI00413451 | 46 kDa | 0.39 | 1 | 1 | 2.76 | 1 | 1.06 | 1 |
| 349 | GRHPR Glyoxylate reductase/hydroxypyruvate reductase | IPI00037448 | 36 kDa | 0.32 | 2.01 | 1 | 1 | 1 | 1.06 | 1 |
| 350 | VPS35 Vacuolar protein sorting-associated protein 35 | IPI00018931 | 92 kDa | 0.86 | 2.01 | 1 | 1.38 | 1 | 2.11 | 1 |
| 351 | PSMA3 Isoform 2 of Proteasome subunit alpha type-3 | IPI00171199 | 28 kDa | 0.13 | 1 | 2.25 | 2.76 | 1 | 1 | 1 |
| 352 | KRT35 keratin 35 | IPI00294649 | 50 kDa | 0.26 | 38.16 | 1 | 39.96 | 1 | 22.21 | 1 |
| 353 | PSMB8 Isoform 1 of Proteasome subunit beta type-8 | IPI00000783 | 30 kDa | 0.27 | 1 | 1 | 1 | 1 | 2.11 | 1.58 |
| 354 | HADHA Trifunctional enzyme subunit alpha, mitochondrial | IPI00031522 | 83 kDa | 0.13 | 2.01 | 1.13 | 1.38 | 1 | 1 | 1 |
| 355 | RPS3 40S ribosomal protein S3 | IPI00011253 | 27 kDa | 0.29 | 1 | 3.38 | 1.38 | 1 | 1 | 1 |
| 356 | SNRPD3 Small nuclear ribonucleoprotein Sm D3 | IPI00017964 | 14 kDa | 0.65 | 1 | 2.25 | 1 | 1 | 1 | 1.58 |
| 357 | ##IPI00555812 | ##IPI00555812 | ? | 0.35 | 1 | 1 | 1 | 1 | 1.06 | 2.37 |
| 358 | GSTM2 Glutathione S-transferase Mu 2 | IPI00219067 | 26 kDa | 0.21 | 1 | 4.5 | 6.89 | 1 | 1 | 1 |
| 359 | KRT34 keratin 34 | IPI00292715 | 49 kDa | 0.38 | 43.18 | 1 | 1 | 1 | 1 | 1 |
| 360 | SERPING1 Plasma protease C1 inhibitor | IPI00291866 | 55 kDa | 0.46 | 1 | 1 | 1 | 3.39 | 1 | 1 |
| 361 | KRT83 Keratin type II cuticular Hb3 | IPI00297795 | 54 kDa | 0.31 | 140.57 | 1 | 1 | 1 | 1 | 1 |
| 362 | HSPG2 Basement membrane-specific heparan sulfate proteoglycan core protein | IPI00024284 | 469 kDa | 0.21 | 2.01 | 1 | 1.38 | 1 | 1.06 | 1 |
| 363 | A26C1A Isoform 1 of ANKRD26-like family C member 1A | IPI00479743 | 121 kDa | 0.0029 | 1 | 61.91 | 59.25 | 25.44 | 1 | 1 |
| 364 | SLC25A5 ADP/ATP translocase 2 | IPI00007188 | 33 kDa | 0.37 | 1 | 2.25 | 1 | 1 | 1 | 1 |
| 365 | THBS1 Thrombospondin-1 | IPI00296099 | 129 kDa | 0.37 | 2.01 | 1 | 1 | 1 | 1 | 1 |
| 366 | ##IPI00658151 | ##IPI00658151 | ? | 0.38 | 1 | 1 | 1 | 1.7 | 1 | 1 |
| 367 | PSME2 Uncharacterized protein PSME2 | IPI00384051 | 29 kDa | 0.65 | 1 | 1 | 1 | 1.7 | 1 | 1 |
| 368 | ##IPI00064607 | ##IPI00064607 | ? | 0.37 | 1 | 2.25 | 1 | 1 | 1 | 1 |
| 369 | ##IPI00005826 | ##IPI00005826 | ? | 0.32 | 2.01 | 1 | 1 | 1 | 1.06 | 1 |
| 370 | WDR1 Isoform 2 of WD repeat-containing protein 1 | IPI00216256 | 58 kDa | 0.38 | 1 | 3.38 | 1 | 1 | 1.06 | 1 |
| 371 | PDIA6 Isoform 2 of Protein disulfide-isomerase A6 | IPI00299571 | 54 kDa | 0.13 | 1 | 2.25 | 2.76 | 1 | 1 | 1 |
| 372 | IGHM IGHM protein | IPI00472610 | 53 kDa | 0.81 | 1 | 109.19 | 1 | 150.09 | 1 | 1 |
| 373 | S100A8 Protein S100-A8 | IPI00007047 | 11 kDa | 0.39 | 1 | 1.13 | 1 | 2.54 | 1 | 1 |
| 374 | GLUD1 Glutamate dehydrogenase 1, mitochondrial | IPI00016801 | 61 kDa | 0.94 | 1 | 2.25 | 1 | 1 | 2.11 | 1 |
| 375 | HNRNPA3 Isoform 1 of Heterogeneous nuclear ribonucleoprotein A3 | IPI00419373 | 40 kDa | 0.42 | 1 | 1.13 | 1 | 1 | 1 | 2.37 |
| 376 | ##IPI00328754 | ##IPI00328754 | ? | 0.37 | 1 | 1 | 1 | 1 | 3.17 | 1 |
| 377 | PYGL Glycogen phosphorylase, liver form | IPI00783313 | 97 kDa | 0.37 | 1 | 3.38 | 1 | 1 | 1 | 1 |
| 378 | ATP2B3 Isoform XB of Plasma membrane calcium-transporting ATPase 3 | IPI00003831 | 134 kDa | 0.95 | 2.01 | 1 | 1 | 1 | 2.11 | 1 |
| 379 | CALR Calreticulin | IPI00020599 | 48 kDa | 0.13 | 2.01 | 2.25 | 1 | 1 | 1 | 1 |
| 380 | CELSR2 Cadherin EGF LAG seven-pass G-type receptor 2 | IPI00015346 | 317 kDa | 0.37 | 1 | 1 | 4.13 | 1 | 1 | 1 |
| 381 | COMP Cartilage oligomeric matrix protein | IPI00028030 | 83 kDa | 0.37 | 3.01 | 1 | 1 | 1 | 1 | 1 |
| 382 | KRT33B Keratin, type I cuticular Ha3-II | IPI00031423 | 46 kDa | 0.24 | 85.35 | 1 | 1 | 1 | 1 | 1 |
| 383 | ELAVL1 cDNA FLJ60076, highly similar to ELAV-like protein 1 | IPI00301936 | 39 kDa | 0.35 | 4.02 | 1 | 1 | 1 | 1 | 1 |
| 384 | BLMH Bleomycin hydrolase | IPI00219575 | 53 kDa | 0.37 | 1 | 1 | 1 | 1 | 1 | 3.16 |
| 385 | FMOD Fibromodulin | IPI00000860 | 43 kDa | 0.37 | 1 | 1 | 1 | 1 | 1 | 3.16 |
| 386 | RHOA Transforming protein RhoA | IPI00478231 | 22 kDa | 0.34 | 1 | 1 | 2.76 | 1 | 1 | 1 |
| 387 | FHL1 Four and a half LIM domains 1 variant | IPI00014398 | 34 kDa | 0.32 | 1 | 1 | 1 | 1.7 | 1.06 | 1 |
| 388 | EZR Ezrin | IPI00746388 | 69 kDa | 0.68 | 1 | 1 | 8.27 | 1 | 4.23 | 1 |
| 389 | ##IPI00175649 | ##IPI00175649 | ? | 0.31 | 1 | 1 | 1 | 1 | 1.06 | 1.58 |
| 390 | GSTK1 Glutathione S-transferase kappa 1 | IPI00219673 | 25 kDa | 0.23 | 1 | 2.25 | 1.38 | 1 | 1 | 1 |
| 391 | XRCC5 ATP-dependent DNA helicase 2 subunit 2 | IPI00220834 | 83 kDa | 0.23 | 1 | 2.25 | 1.38 | 1 | 1 | 1 |
| 392 | TGM3 Protein-glutamine gamma-glutamyltransferase E | IPI00300376 | 77 kDa | 0.72 | 1 | 1 | 1 | 1 | 1 | 1.58 |
| 393 | ##IPI00398505 | ##IPI00398505 | ? | 0.47 | 1 | 1 | 1 | 1.7 | 1 | 1 |
| 394 | ZNF337 Zinc finger protein 337 | IPI00000110 | 87 kDa | 0.37 | 1 | 2.25 | 1 | 1 | 1 | 1 |
| 395 | RYR2 Isoform 1 of Ryanodine receptor 2 | IPI00023217 | 565 kDa | 0.37 | 1 | 1 | 2.76 | 1 | 1 | 1 |
| 396 | COL16A1 Isoform 1 of Collagen alpha-1(XVI) chain | IPI00400935 | 158 kDa | 0.57 | 1 | 1 | 1 | 1 | 2.11 | 1 |
| 397 | CS Citrate synthase, mitochondrial | IPI00025366 | 52 kDa | 0.37 | 1 | 2.25 | 1 | 1 | 1 | 1 |
| 398 | USP37 Ubiquitin carboxyl-terminal hydrolase 37 | IPI00472977 | 110 kDa | 0.30 | 2.01 | 1 | 1 | 1 | 1 | 1 |
| 399 | AZGP1 alpha-2-glycoprotein 1, zinc | IPI00166729 | 34 kDa | 0.57 | 1 | 1 | 1 | 1 | 1 | 2.37 |
| 400 | ERAP1 Isoform 2 of Endoplasmic reticulum aminopeptidase 1 | IPI00165949 | 108 kDa | 0.37 | 3.01 | 1 | 1 | 1 | 1 | 1 |
| 401 | ZNF646 Zinc finger protein 646 | IPI00004419 | 201 kDa | 0.37 | 1 | 1 | 2.76 | 1 | 1 | 1 |
| 402 | SHROOM4 Isoform 1 of Protein Shroom4 | IPI00008176 | 165 kDa | 0.37 | 1 | 1 | 1 | 1 | 1 | 1.58 |
| 403 | FAM117A Protein FAM117A | IPI00006580 | 48 kDa | 0.37 | 2.01 | 1 | 1 | 1 | 1 | 1 |
| 404 | FABP4 Fatty acid-binding protein, adipocyte | IPI00215746 | 15 kDa | 0.49 | 1 | 1 | 1 | 1 | 3.17 | 1 |
| 405 | DLST Full-length cDNA 5-PRIME end of clone CS0DB006YE12 of Neuroblastoma of Homo sapiens | IPI00384122 | 39 kDa | 0.29 | 1 | 3.38 | 1 | 1 | 1 | 1 |
| 406 | LIMS2 Isoform 2 of LIM and senescent cell antigen-like-containing domain protein 2 | IPI00398576 | 42 kDa | 0.38 | 1 | 1 | 4.13 | 1 | 1 | 1 |
| 407 | PIR Pirin | IPI00012575 | 32 kDa | 0.38 | 1 | 3.38 | 1 | 1 | 1 | 1 |
| 408 | KLK7 Isoform 1 of Kallikrein-7 | IPI00028600 | 28 kDa | 0.57 | 1 | 1 | 1 | 1 | 1 | 2.37 |
| 409 | ##IPI00020701 | ##IPI00020701 | ? | 0.29 | 1 | 3.38 | 1 | 1 | 1 | 1 |
| 410 | ##IPI00007834 | ##IPI00007834 | ? | 0.37 | 1 | 1 | 1 | 1 | 1 | 1.58 |
| 411 | ##IPI00011286 | ##IPI00011286 | ? | 0.23 | 1 | 2.25 | 1 | 1 | 1 | 1 |
| 412 | ##IPI00025702 | ##IPI00025702 | ? | 0.13 | 2.01 | 1 | 1 | 1 | 1 | 1 |
| 413 | IDH2 Isocitrate dehydrogenase [NADP], mitochondrial | IPI00011107 | 51 kDa | 0.37 | 2.01 | 1 | 1 | 1 | 1 | 1 |
| 414 | MT-CO2 Cytochrome c oxidase subunit 2 | IPI00017510 | 26 kDa | 0.37 | 1 | 1 | 1 | 1 | 2.11 | 1 |
| 415 | ABHD10 Abhydrolase domain-containing protein 10, mitochondrial | IPI00020075 | 34 kDa | 0.39 | 1 | 2.25 | 1 | 1 | 1 | 1 |
| 416 | RRAS Ras-related protein R-Ras | IPI00020418 | 23 kDa | 0.43 | 1 | 1 | 1 | 1 | 2.11 | 1 |
| 417 | APOE Apolipoprotein E | IPI00021842 | 36 kDa | 0.38 | 1 | 1 | 1 | 1.7 | 1 | 1 |
| 418 | CNDP2 PP856 | IPI00165579 | 44 kDa | 0.37 | 2.01 | 1 | 1 | 1 | 1 | 1 |
| 419 | PDCD6IP PDCD6IP protein | IPI00246058 | 97 kDa | 0.19 | 2.01 | 1 | 1 | 1 | 1 | 1 |
| 420 | MLL3 Isoform 1 of Histone-lysine N-methyltransferase MLL3 | IPI00168806 | 541 kDa | 0.37 | 1 | 2.25 | 1 | 1 | 1 | 1 |
| 421 | - Ig kappa chain V-I region Ni | IPI00387106 | 12 kDa | 0.32 | 1 | 1 | 1 | 1.7 | 1 | 1 |
| 422 | CTNNA1 Isoform 1 of Catenin alpha-1 | IPI00215948 | 100 kDa | 0.37 | 2.01 | 1 | 1 | 1 | 1 | 1 |
| 423 | DDX58 Isoform 2 of Probable ATP-dependent RNA helicase DDX58 | IPI00295503 | 101 kDa | 0.37 | 1 | 2.25 | 1 | 1 | 1 | 1 |
| 424 | PRKAR2A PRKAR2A protein | IPI00063234 | 43 kDa | 0.23 | 1 | 2.25 | 1 | 1 | 1 | 1 |
| 425 | ##IPI00181670 | ##IPI00181670 | ? | 0.23 | 1 | 2.25 | 1 | 1 | 1 | 1 |
| 426 | ##IPI00396279 | ##IPI00396279 | ? | 0.72 | 1 | 1 | 1 | 1 | 1 | 1.58 |
| 427 | GRIA3 Isoform Flip of Glutamate receptor 3 | IPI00219315 | 101 kDa | 0.39 | 1 | 2.25 | 1 | 1 | 1 | 1 |
| 428 | ##IPI00163920 | ##IPI00163920 | ? | 0.43 | 1 | 1 | 1 | 1 | 2.11 | 1 |
| 429 | ##IPI00044326 | ##IPI00044326 | ? | 0.57 | 1 | 1 | 1 | 1 | 2.11 | 1 |
| 430 | MYO15A Myosin-XV | IPI00152380 | 395 kDa | 0.37 | 1 | 2.25 | 1 | 1 | 1 | 1 |
| 431 | TTC6 Tetratricopeptide repeat protein 6 | IPI00244812 | 59 kDa | 0.63 | 1 | 1 | 1 | 1 | 2.11 | 1 |
| 432 | ##IPI00176709 | ##IPI00176709 | ? | 0.40 | 2.01 | 1 | 1 | 1 | 1 | 1 |
| 433 | COLEC12 Isoform 1 of Collectin-12 | IPI00414467 | 82 kDa | 0.38 | 1 | 1 | 1 | 1 | 2.11 | 1 |
| 434 | CCT6A T-complex protein 1 subunit zeta | IPI00027626 | 58 kDa | 0.40 | 2.01 | 1 | 1 | 1 | 1 | 1 |
| 435 | ##IPI00478772 | ##IPI00478772 | ? | 0.82 | 1 | 1 | 1 | 1.7 | 1 | 1 |
| 436 | TMEM43 Transmembrane protein 43 | IPI00301280 | 45 kDa | 0.23 | 1 | 2.25 | 1 | 1 | 1 | 1 |
| 437 | HDAC1 Histone deacetylase 1 | IPI00013774 | 55 kDa | 0.37 | 1 | 1 | 2.76 | 1 | 1 | 1 |
| 438 | MAP1B Microtubule-associated protein 1B | IPI00008868 | 271 kDa | 0.37 | 1 | 1 | 1 | 1 | 1 | 1.58 |

^1^ International Protein Index access number, ^2^ p-value

MS searching info.

Fragment Tolerance: 1.00 Da (Average)

Parent Tolerance: 1.5 Da (Average)

Fixed Modifications:

Variable Modifications: +16 on M (Oxidation), +57 on C (Carbamidomethyl)

Database: the ipi.HUMAN.v3.49 database (unknown version, 74017 entries)

Digestion Enzyme: Trypsin

Max Missed Cleavages: 2
