# Supplementary material for: Human giant congenital melanocytic nevus exhibits potential proteomic alterations leading to melanotumorigenesis
Source: Proteome Sci. 2012 Aug 20;10:50. doi: 10.1186/1477-5956-10-50 (PMC3575290; doi:10.1186/1477-5956-10-50)
Supplement: Additional file 3 — Figure S2. The functional network and enriched functional group of GCMN was analyzed using ClueGO, a biological term enrichment analyzer. The following proteins were significantly enriched in GCMN: melanosome (GO_cellular component), neurotrophin signaling pathway (KEGG pathway), downregulated of MTA-3 in ER-negative breast tumors (Biocarta), cell cycle (KEGG pathway), phospholipase inhibitor activity (GO_molecular function), and glycolysis/gluconeogenesis (KEGG pathway). [file 1477-5956-10-50-S3.pdf]

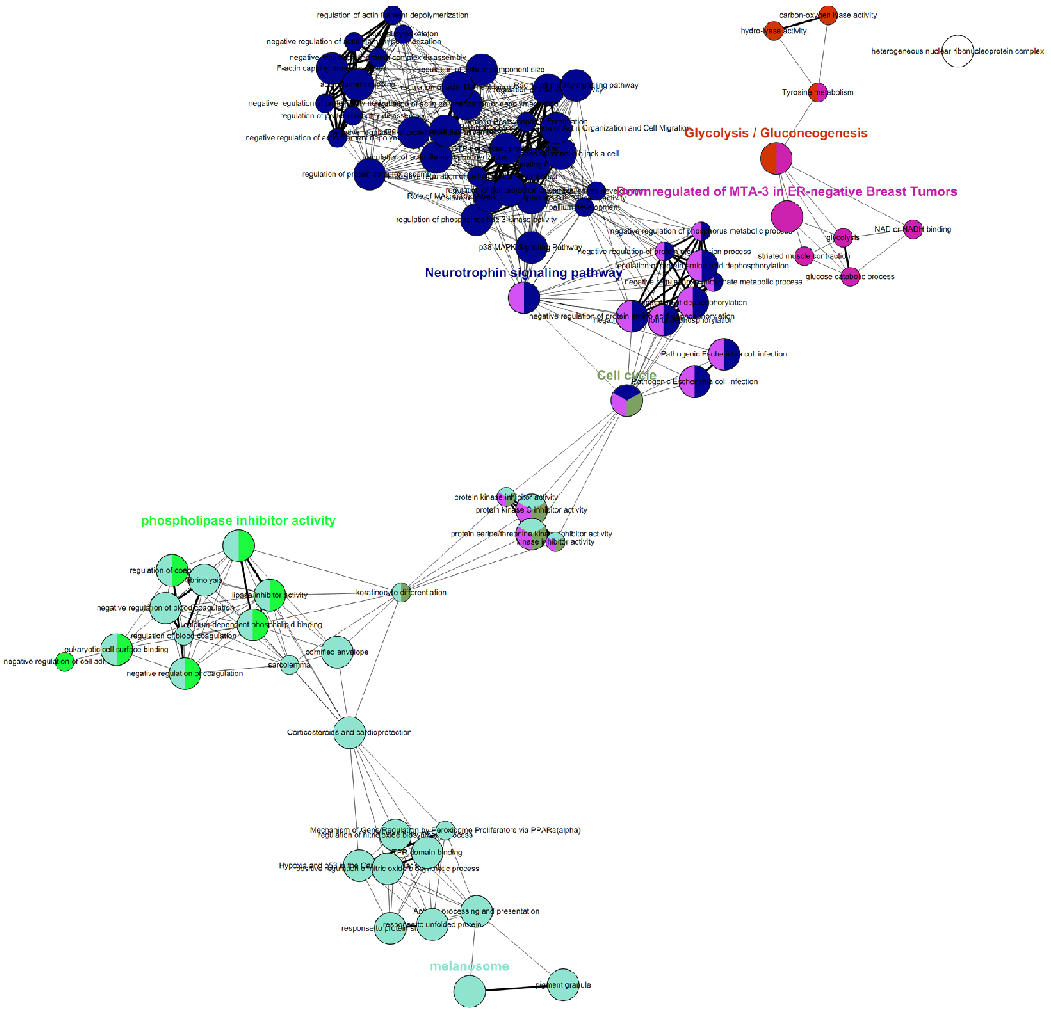

Figure S2. The functional network and enriched functional group of GCMN was analyzed using ClueGO, a biological term enrichment analyzer. The following proteins were significantly enriched in GCMN: melanosome (GO_cellular component), neurotrophin signaling pathway (KEGG pathway), downregulated of MTA-3 in ER-negative breast tumors (Biocarta), cell cycle (KEGG pathway), phospholipase inhibitor activity (GO_molecular function), and glycolysis/gluconeogenesis (KEGG pathway).
