# Supplementary material for: Human giant congenital melanocytic nevus exhibits potential proteomic alterations leading to melanotumorigenesis
Source: Proteome Sci. 2012 Aug 20;10:50. doi: 10.1186/1477-5956-10-50 (PMC3575290; doi:10.1186/1477-5956-10-50)
Supplement: Additional file 4 — Table S2. Integrated densitometry value of Western blot band. [file 1477-5956-10-50-S4.doc]

Table S2. Integrated densitometry value of Western blot band

| ID | 14-3-3 epsilon/GAPDH | 14-3-4 tau/GAPDH | Prohibitin/b-tubulin |
| --- | --- | --- | --- |
| Normal 1 | 2.36 | 0.09 | 0.70 |
| Normal 2 | 1.53 | 0.12 | 0.66 |
| Normal 3 | 1.50 | 0.13 | 0.80 |
| Normal 4 | 1.07 | 0.08 | 0.72 |
| Normal 5 | 0.92 | 0.07 | 0.78 |
| Normal 6 | 2.28 | 0.08 | 0.56 |
| Normal 7 | 1.05 | 0.29 | 0.52 |
| GCMN1 | 2.33 | 0.21 | 0.98 |
| GCMN2 | 2.28 | 0.22 | 1.10 |
| GCMN3 | 3.27 | 0.31 | 0.96 |
| GCMN4 | 3.90 | 0.22 | 1.64 |
| GCMN5 | 1.25 | 0.16 | 1.33 |
| GCMN6 | 1.99 | 0.29 | 3.24 |
| GCMN7 | 2.91 | 0.33 | 1.20 |
