# Supplementary material for: Human giant congenital melanocytic nevus exhibits potential proteomic alterations leading to melanotumorigenesis
Source: Proteome Sci. 2012 Aug 20;10:50. doi: 10.1186/1477-5956-10-50 (PMC3575290; doi:10.1186/1477-5956-10-50)
Supplement: Additional file 5 — Table S3. Statistical analysis result of western blot. [file 1477-5956-10-50-S5.doc]

Table S3. Statistical analysis result of western blot

| Item | Group | 14-3-3 epsilon/GAPDH | 14-3-4 tau/GAPDH | Prohibitin/b-tubulin |
| --- | --- | --- | --- | --- |
| Mean | Normal | 1.53 | 0.12 | 0.68 |
|  | GCMN | 2.56 | 0.25 | 1.49 |
| SEM | Normal | 0.22 | 0.03 | 0.04 |
|  | GCMN | 0.33 | 0.02 | 0.30 |
| student's  t-test | p-value | 0.03 | 0.01 | 0.04 |
